# Multi-Omics Integration Identifies Epithelial-Stromal-Immune Co-Regulators Bridging Ulcerative Colitis and Colorectal Cancer

**DOI:** 10.3390/biomedicines14092120

**Published:** 2026-09-19

**Authors:** Wenhao Sun, Zhiwei Jiang

**Affiliations:** 1 Department of General Surgery, Affiliated Hospital of Nanjing University of Chinese Medicine, Nanjing 210029, China; swh@njucm.edu.cn; 2 The First School of Clinical Medicine, Nanjing University of Chinese Medicine, Nanjing 210023, China

**Keywords:** ulcerative colitis, colorectal cancer, spatial transcriptomics, Mendelian randomization, tumour microenvironment

## Abstract

**Background/Objectives:** Patients with ulcerative colitis (UC) carry a substantially elevated risk of developing colorectal cancer (CRC); yet, the transcriptional intermediates that bridge chronic mucosal inflammation and colorectal malignancy remain poorly characterized. A related question is whether any genes shared between the two conditions actually help drive transformation, or whether they represent downstream responders. **Methods:** We integrated two UC microarray cohorts (totalling 227 samples) and the TCGA-COAD dataset (*n* = 524), combining weighted gene co-expression network analysis (WGCNA) with directional differential expression to define shared candidate genes. Three machine-learning approaches—LASSO, random forest, and SVM-RFE—were applied to that candidate set, and the stability of their intersection was assessed by bootstrap resampling. The resulting panel was then examined by replication in an independent UC cohort, two-sample Mendelian randomization, immune deconvolution, survival modelling, and Visium spatial transcriptomics. **Results:** WGCNA and directional differential expression together yielded 91 shared candidate genes, and the three algorithms converged on four: *CXCL1*, *S100P*, *THY1*, and *TRIM29*. Bootstrap resampling showed that this convergence is a property of one particular fit rather than a reproducible selector (all four recovered together in 0.3% of 1000 resamples), so we treated the ensemble as a hypothesis-generating step and made our case for the panel based on independent downstream evidence. This evidence was consistent, as, in an independent UC cohort (GSE92415, *n* = 183), all four genes moved in the same direction as in discovery, and a four-gene model separated inflamed from control mucosa with a cross-validated AUC of 0.986 (95% CI 0.965–1.000). Two-sample Mendelian randomization using strong blood cis-eQTL instruments (*F* = 345 for *CXCL1*, *F* = 2423 for *S100P*) returned null estimates; for *S100P*, the design had 80% power to detect an odds ratio of 1.07 per standard deviation, so this is an informative null rather than an absence of data, while *THY1* and *TRIM29* had no cis-eQTLs in eQTLGen and remain untestable. Each gene showed a distinct pattern of immune cell correlation in TCGA-COAD. LASSO-Cox retained only *CXCL1*, whose higher expression was associated with a better rather than worse overall survival (HR 0.79 per SD, *p* = 0.020); its discrimination was weak (optimism-corrected C-index 0.56 for the score alone) and did not reach significance in an external cohort (GSE39582, *n* = 573; *p* = 0.11), so we reported no prognostic model. In Visium spatial data from four colonic sections, a three-compartment structure was reproducible between UC sections—*CXCL1* near the neutrophil and interferon signals, *THY1* within the stroma and anticorrelated with the epithelium, and *S100P* and *TRIM29* alongside the epithelium—although, with two sections per group, the difference in module scores between the UC and control mucosa was not resolvable. **Conclusions:** Collectively, these four genes describe a spatially structured epithelial–stromal–immune module of the inflamed mucosa; however, the findings are hypothesis-generating and are not sufficient for prognostic or stratification use.

## 1. Introduction

Ulcerative colitis is a chronic, relapsing inflammatory bowel disease. Its hallmarks are persistent mucosal inflammation, a compromised epithelial barrier, and a progressive breakdown of immune homeostasis [1,2,3]. One of its most serious, long-term consequences is colorectal cancer; depending on the study, the excess risk ranges from two- to eight-fold, and the cumulative incidence has been reported to approach 18% after 30 years of disease [4,5]. This inflammatory trajectory, passing through a dysplasia stage, is generally referred to as colitis-associated colorectal cancer (CAC). Its molecular trajectory is still only partly understood [6,7]. Unlike the situation in sporadic CRC, where a handful of well-characterized driver mutations tend to dominate, CAC looks more like a slow, chronic remodelling of the microenvironment. We reason that repeated cycles of epithelial injury and repair gradually remodel the epithelium, stroma, and immune compartments into a state that collectively becomes permissive for tumour growth. Under this model, the molecular bridge between inflammation and cancer is not a novel oncogene awaiting discovery but a set of regulatory molecules jointly mobilized by the shared inflammatory milieu of UC and CRC.

Most transcriptomic work to date has kept the two diseases in separate lanes. Researchers have focused either on inflammatory signatures in the UC mucosa or on the heterogeneity of the CRC microenvironment. As a consequence, a systematic molecular picture of what links the two has remained elusive [8,9]. More recently, groups have started trying to bridge this gap. One approach that has shown promise is to look for shared genes between UC and CRC, sometimes with the backing of computational findings from Mendelian randomization, as Yu and colleagues did when they put forward *MMP1* as a candidate diagnostic marker from intersecting UC-CRC gene sets [10]. Studies like these support the general premise that shared regulators exist between the two diseases. A common limitation, however, is that most studies stop once they have obtained a gene list. Two questions tend to be left hanging: which cellular compartment does each gene actually belong to, and is it a causal driver or merely a downstream responder? Until we can answer both, it is hard to say whether a shared gene represents something that can be acted on mechanistically.

Tackling both questions at the same time calls for evidence from multiple layers, each with its own strengths and blind spots. WGCNA picks up co-regulated gene modules without any prior assumptions [11], while ensemble machine learning helps sift through those modules to find the most robust features. Two-sample MR goes a step further: it uses germline genetic variants as instruments to nudge the association evidence one notch closer to causation [12]. And spatial transcriptomics puts genes back into their actual cellular neighbourhoods, which offers a much more direct separation of the epithelium, stroma, and immune infiltrate than the computational deconvolution of bulk RNA-seq. None of these methods are perfect on their own, but, when their results converge on the same answer, that answer becomes considerably more believable than that which any single analysis could deliver.

We hypothesized that a limited set of immune-microenvironment genes is co-regulated across UC and CRC and participates in the inflammation-to-cancer transition along three axes: epithelial, stromal and immune. Two design decisions followed from the driver-versus-mediator distinction. First, MR was deployed as a formal test of whether the germline variation in the expression of the nominated genes affects the CRC risk. It is worth being precise about what this can and cannot show: MR interrogates the lifelong, genetically determined expression, whereas the biology we are proposing is the inflammation-induced expression in the mucosa. A null MR estimate therefore bears on the first question and not the second, and we interpret it accordingly. Second, spatial transcriptomics was incorporated to assign each gene to a physical tissue compartment, giving the three-axis model a morphological reference that bulk correlation alone cannot supply. Because the spatial dataset comprises only four sections, we present it as exploratory mapping rather than as validation.

## 2. Materials and Methods

### 2.1. Data Sources

Two UC mucosal microarray datasets were retrieved from the Gene Expression Omnibus. GSE87466 was run on the Affymetrix HT HG-U133+ PM platform and contains 87 UC samples and 21 normal controls. GSE75214 used the Affymetrix Human Gene 1.0 ST array and includes 97 samples from patients with active UC alongside 22 normal controls. The TCGA-COAD cohort, comprising 483 tumour specimens and 41 matched normal samples, was downloaded via TCGAbiolinks using the STAR-Counts workflow [13]. For the spatial transcriptomic component, GSE189184 was used and four tissue sections were analysed: B10 and C5 served as healthy controls, and B5 and B12 represented inflamed UC mucosa [14]. Cis-eQTL data were obtained from the eQTLGen consortium (whole blood, *n* = 31,684) [15], and CRC GWAS summary statistics for participants of European ancestry (6581 cases and 463,421 controls) were taken from the cross-population association atlas of Sakaue et al. [16], accessed through the IEU OpenGWAS database under accession ebi-a-GCST90018808.

### 2.2. Bulk Transcriptome Preprocessing

Probes were mapped to gene symbols using the manufacturer’s annotation files (GPL13158 for GSE87466 and GPL6244 for GSE75214). Where a transcript mapped to more than one gene, the first symbol listed in the gene_assignment field was retained. Probes mapping to the same gene were collapsed with limma::avereps. After the two UC matrices were merged on 16,595 shared gene symbols, batch effects were removed with sva::ComBat, retaining disease status as a biological covariate. For the CRC data, the 60,660 raw counts from TCGA-COAD underwent variance-stabilizing transformation in DESeq2. Ensembl IDs were converted to gene symbols with org.Hs.eg.db, and, where several Ensembl IDs mapped to the same symbol, the transcript with the strongest differential signal was retained.

### 2.3. Differential Expression and Network Analysis

Differential expression analysis used limma for the UC data and DESeq2 for the CRC data, both at a threshold of |log_2_FC| > 1 and adjusted *p* < 0.05. Directionally concordant shared DEGs were defined as genes whose expression changed in the same direction in both diseases, either up in both or down in both. WGCNA (version 1.72) was run separately on each disease dataset, with input restricted to the 5000 most variable genes in each case. Soft thresholds of 12 for UC and 7 for CRC were chosen on the basis of scale-free topology model fits (*R*^2^ > 0.65 and >0.85, respectively). Modules were called by dynamic tree cutting with a minimum module size of 50 genes and merged at a cut height of 0.25, and module eigengenes were correlated against the binary disease trait (Supplementary Figure S1).

### 2.4. Ensemble Feature Selection

All genes belonging to trait-associated modules were pooled within each disease: the green and turquoise modules in UC, and the yellow, turquoise, black, blue, pink, and green modules in CRC. The intersection of these two pools yielded 919 shared module genes, and intersecting these with the 311 directionally concordant DEGs left 91 candidates. Three feature-selection algorithms were then applied to the UC expression matrix. LASSO logistic regression was run with 10-fold cross-validation and the penalty was selected at lambda.1se (Supplementary Figure S2). Random forest was run with ntree = 500, and the 20 genes with the highest mean decrease in Gini were taken. Linear-kernel SVM-RFE was implemented with the e1071 package, again retaining 20 features. Only genes that appeared in all three lists were carried into the downstream analysis.

### 2.5. Stability, Permutation, and External Validation of the Gene Panel

Three analyses were added during revision to characterize the robustness of the panel. First, the entire feature-selection pipeline was repeated in 1000 bootstrap resamples, drawn with replacement within each disease group so as to preserve the UC-to-control ratio; for each resample, LASSO, random forest, and SVM-RFE were refitted with the hyperparameters used in the primary analysis and the selection frequency of every candidate gene was recorded, defined as the proportion of resamples in which it appeared in all three lists. The same pipeline was rerun in GSE87466 and GSE75214 separately. Because the SVM-RFE implementation used in the primary analysis was not designed for repeated refitting, resampling used a standard linear-kernel SVM-RFE that eliminates the lowest-weight 10% of features per iteration down to 20 features; applied to the full cohort, this reproduced the original four-gene intersection exactly. Compartment membership was assigned by correlating each of the 91 candidates with the four hub genes and taking the nearest, and an independent set of 500 resamples was used to quantify how often each compartment was represented. Second, disease labels were permuted 1000 times and the pipeline was rerun to obtain a null distribution for the size of the three-way intersection. Third, the panel was tested in two datasets that did not contribute to discovery: GSE92415 for differential expression in UC mucosa, summarized by per-gene AUC and by the cross-validated AUC of a four-gene logistic model with confidence intervals from 2000 bootstrap resamples, and GSE39582 for the survival analysis described above.

### 2.6. Two-Sample Mendelian Randomization

To evaluate potential upstream causality, we performed two-sample MR using cis-eQTLs as genetic instruments for each hub gene. Cis-eQTLs within ±1 Mb of the transcription start site were extracted from eQTLGen (FDR < 0.05). *Z*-scores were converted to effect estimates: β = *Z*/√*N* and SE = 1/√*N*. Genome-wide significant variants (*p* < 5 × 10^−8^) were clumped at *r*^2^ < 0.001 within a 10 Mb window, using the 1000 Genomes European panel as the LD reference. Exposure and outcome data were harmonized in the TwoSampleMR package (v0.6.0). We estimated causal effects with five methods: IVW, MR-Egger, weighted median, simple mode and weighted mode. Heterogeneity was assessed by Cochran’s Q test, horizontal pleiotropy by the MR-Egger intercept, and the influence of individual variants by leave-one-out analysis. Instrument strength was quantified per SNP as *R*^2^ = *Z*^2^/(*N* + *Z*^2^), which avoids reliance on allele frequencies that eQTLGen does not report, and as *F* = *R*^2^(*N* − 2)/(1 − *R*^2^); the overall F statistic was computed as *R*^2^(*N* − *k* − 1)/[*k*(1 − *R*^2^)]. Statistical power was calculated following Brion and colleagues for a binary outcome, given the observed *R*^2^, and the 6581 cases and 463,421 controls of the outcome GWAS and α = 0.05, and is reported as the minimum odds ratio detectable at 80% power. Directionality was tested with MR-Steiger. For the two genes without genome-wide significant instruments, we additionally examined the full eQTLGen cis-eQTL table to determine whether any variant reached FDR < 0.05 at all.

### 2.7. Survival Modelling, Immune Infiltration, and Drug-Pathway Analysis

In the 459 TCGA-COAD tumour samples with survival annotation (97 deaths), we derived a risk score of the form RS = Σβ_i_ × Exp_i_ using LASSO-Cox regression over the four hub genes with 10-fold cross-validation. Only *CXCL1* retained a non-zero coefficient, so the resulting score is a linear function of that gene; we report the number of retained genes rather than describing it as a four-gene score. Patients were split at the median score and compared by the Kaplan–Meier method with a log-rank test. Time-dependent ROC curves were generated with the timeROC package. To test whether the score carried independent prognostic information, we fitted a multivariable Cox model including age in years, sex, and AJCC stage (dichotomized as I–II versus III–IV); hazard ratios are reported both per unit and per standard deviation, since the per-unit scale of a LASSO-Cox score is arbitrary. The proportional-hazards assumption was checked with scaled Schoenfeld residuals, globally and per covariate. Model discrimination was corrected for optimism by 1000 bootstrap resamples of the full modelling procedure (rms::validate) and, independently, by 10 repeats of 10-fold cross-validation; apparent and corrected C-indices are both reported, for the full model and for the score alone. A nomogram and bootstrap calibration curves (rms package) were built on the complete-case subset (*n* = 279, 54 deaths). External validation used GSE39582 (*n* = 573 with survival data, 190 deaths): expression was standardized within the cohort, the TCGA-derived coefficient was applied, and the score was tested by Cox regression, alongside each gene individually. Immune infiltration was estimated by ssGSEA using the 12 immune cell type marker sets from Charoentong et al. [17] and the IOBR package, computed with the GSVA algorithm. Spearman correlations were then calculated between each hub gene’s expression and these immune scores, as well as with the expression of nine checkpoint genes (*CD274*, *PDCD1*, *CTLA4*, *HAVCR2*, *LAG3*, *TIGIT*, *PDCD1LG2*, *IDO1*, and *SIGLEC15*) and with pathway signature scores for six CRC therapies: 5-fluorouracil, oxaliplatin, irinotecan, cetuximab, bevacizumab, and pembrolizumab. These signature scores describe transcriptional pathway activity, not clinical response, which was not available for this cohort.

### 2.8. Spatial Transcriptomic Analysis

Space Ranger output files were loaded into Seurat (v5.0) with Load10X_Spatial. Spots were filtered to retain those with nCount > 500, nFeature > 200, and a mitochondrial read fraction below 25% (Supplementary Figure S6). Each section was normalized independently with SCTransform. The four-gene module score for each spot was defined as the arithmetic mean of the SCT-normalized expression values of *CXCL1*, *S100P*, *THY1*, and *TRIM29*. For the co-localization analysis, the two UC sections were merged and jointly renormalized. Cell-type module scores for neutrophil, CAF, regulatory T cell, macrophage, epithelium, and interferon response were computed from their respective canonical marker gene sets, and Spearman correlations were then calculated between the spot-level expression of each hub gene and each cell-type module score across all 1516 UC spots. Spatial clustering was performed on 30 principal components with FindClusters at a resolution of 0.5. Because spots from the same section are not independent observations, all group comparisons were additionally performed at the section level: spot-level values were aggregated into section pseudobulk means and compared descriptively, and the spot-level comparison was refitted as a linear mixed model of the form expression ~ group + (1|section), the lme4 formula notation for a random intercept for section, using lme4 with Satterthwaite degrees of freedom from lmerTest. Co-localization coefficients were also recomputed separately within each UC section, and consistency of sign between the two sections, rather than the *p* value obtained from the merged spot pool, was used as the criterion for reporting a spatial association.

### 2.9. Statistical Analysis

All analyses were conducted in R version 4.5.2, with the analyses added during revision run in R 4.6.0. Two-sided tests were used throughout, with *p* < 0.05 as the significance threshold and Benjamini–Hochberg correction applied wherever multiple testing was a concern. Continuous variables are reported as median and interquartile range and were compared with the Wilcoxon rank-sum test. Where observations were clustered within a sample or a tissue section, inference was based on the sample or section rather than on the individual observation; naive spot-level *p* values, where shown, are identified as such in the corresponding figure legends. Effect sizes with confidence intervals are reported alongside *p* values throughout. All analyses added during revision—the instrument-strength and power calculations, the bootstrap stability and permutation analyses, the optimism-corrected discrimination estimates, the mixed-model spatial reanalysis, and the two external validation cohorts—were not pre-specified and are identified as post hoc.

## 3. Results

### 3.1. Harmonization of Two UC Cohorts Across 16,595 Shared Genes

**Figure 1:**
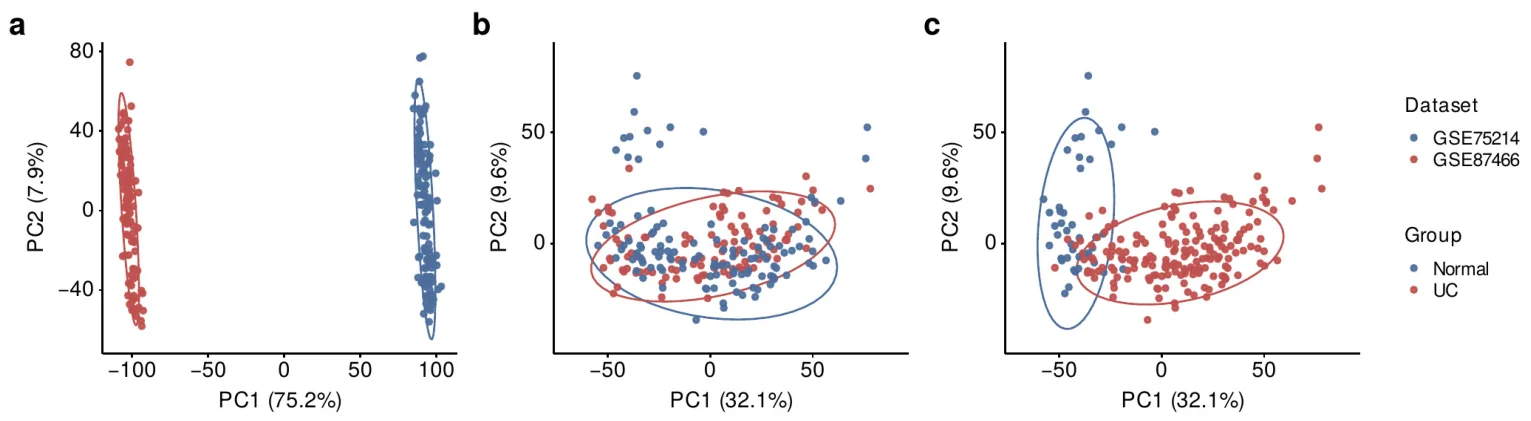
Principal component analysis of the UC bulk transcriptome before and after batch correction. (**a**) Before correction, samples separate along PC1 by study of origin (GSE87466, *n* = 108; GSE75214, *n* = 119). (**b**) After ComBat correction, the two datasets intermix. (**c**) The corrected data coloured by disease status, showing that the separation between UC and control mucosa is preserved. Points are coloured by study of origin in (**a**,**b**) and by disease status in (**c**), following the legends at the right; the two legends apply to all three panels. Ellipses enclose 90% of the samples in each group, and axis labels give the proportion of variance explained.

The two UC microarray datasets were merged across the 16,595 gene symbols present on both platforms, yielding 108 samples from one study and 119 from the other, 227 in total. Before correction, principal component analysis separated the samples clearly by study of origin. After ComBat correction, this batch-driven structure disappeared, whereas the variance attributable to disease status was preserved (Figure 1). This harmonized expression matrix was used as the common starting point for both the differential expression analysis and WGCNA.

### 3.2. Definition of 91 Shared Candidates by Differential Expression and WGCNA

**Figure 2:**
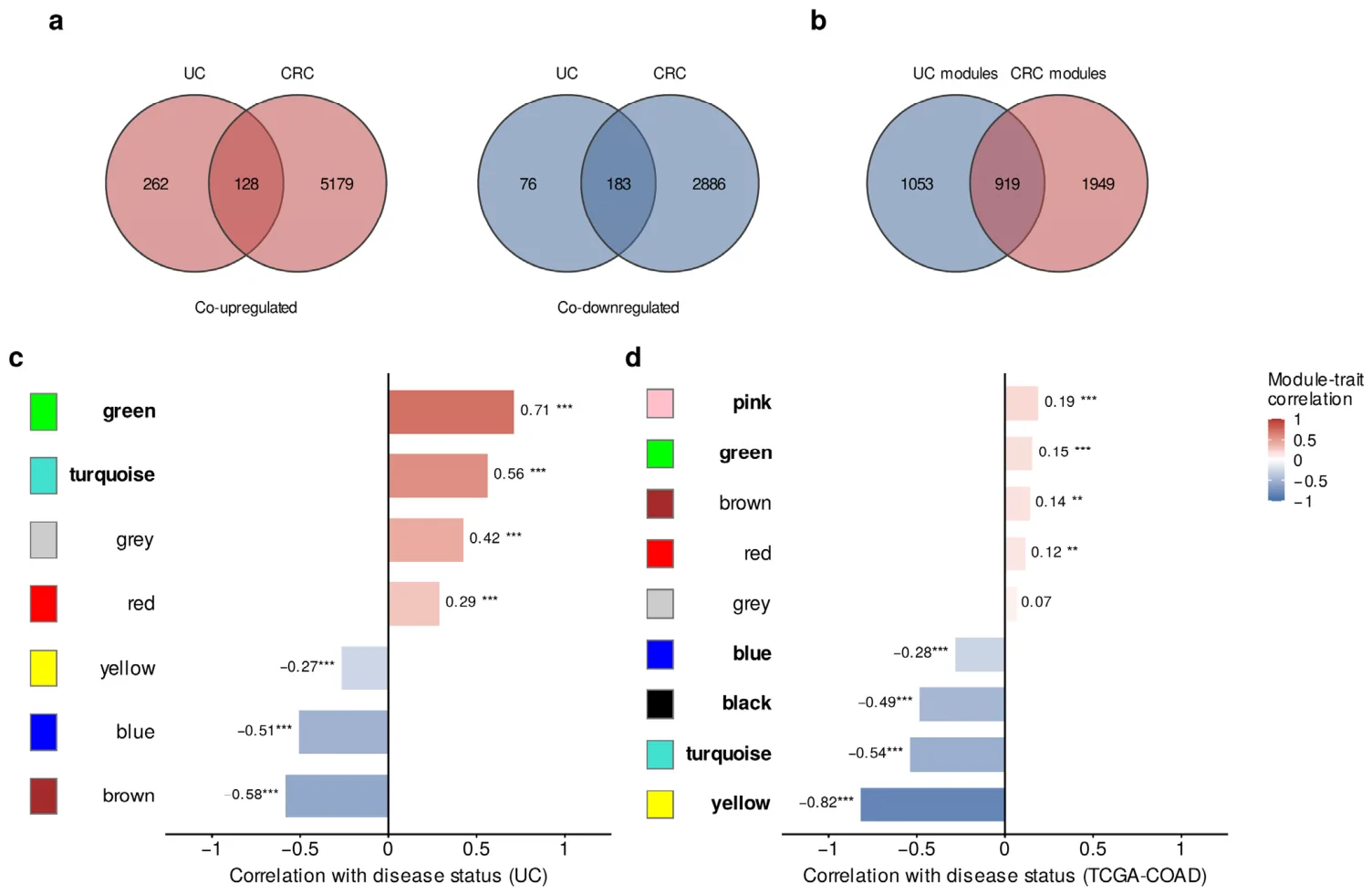
Shared transcriptional programmes across UC and CRC. (**a**) Venn diagrams of directionally concordant differentially expressed genes: 128 co-upregulated (left) and 183 co-downregulated (right). (**b**) Venn diagram of the overlap among trait-associated module genes, totalling 919 shared module genes. (**c**) Correlation between each WGCNA module eigengene and disease status in the harmonized UC cohort. (**d**) The same analysis in TCGA-COAD. The coloured square beside each row identifies the WGCNA module; bar length and the adjacent value give the module-trait correlation, with asterisks for the associated *p* value (** *p* < 0.01 and *** *p* < 0.001). Module names in bold were carried forward into the intersection analysis. The grey module contains genes not assigned to any module and is shown for completeness only.

limma applied to the harmonized UC matrix identified 649 differentially expressed genes (DEGs), 390 upregulated and 259 downregulated. In parallel, DESeq2 applied to the TCGA-COAD data identified 8376 DEGs, 5307 upregulated and 3069 downregulated. Across these two sets, 311 genes changed in the same direction in both diseases: 128 were co-upregulated and 183 co-downregulated (Figure 2a). In the network analysis, WGCNA resolved seven co-expression modules in UC, two of which were strongly associated with disease status: the green module (correlation with disease status, *r* = 0.71; *p* = 2 × 10^−36^) and the turquoise module (*r* = 0.56; *p* = 2 × 10^−20^), both shifted towards UC (Figure 2c). Nine modules were obtained in the TCGA-COAD data, six of which showed significant trait associations (Figure 2d). All genes belonging to trait-associated modules were pooled within each disease and the two pools were intersected, producing 919 shared module genes (Figure 2b). Intersecting these 919 genes with the 311 directionally concordant DEGs yielded 91 high-confidence shared candidates. The resulting list includes genes previously implicated in the inflammation–cancer axis: classical chemokines (*CXCL1*, *CXCL2*, *CXCL3*, *CXCL5*, *CXCL6*, *CXCL10*, *CXCL11*, *CCL3*, *CCL18*, and *CCL20*), matrix-remodelling factors (*COL1A1*, *COL1A2*, *COL3A1*, *MMP1*, *MMP3*, *MMP9*, *MMP12*, and *FAP*), and a group of inflammatory mediators including *IL1B*, *IL1RN*, *TREM1*, *PTGS2*, *SPP1*, and *S100P*.

### 3.3. Convergence of Three Feature-Selection Algorithms on Four Hub Genes

**Figure 3:**
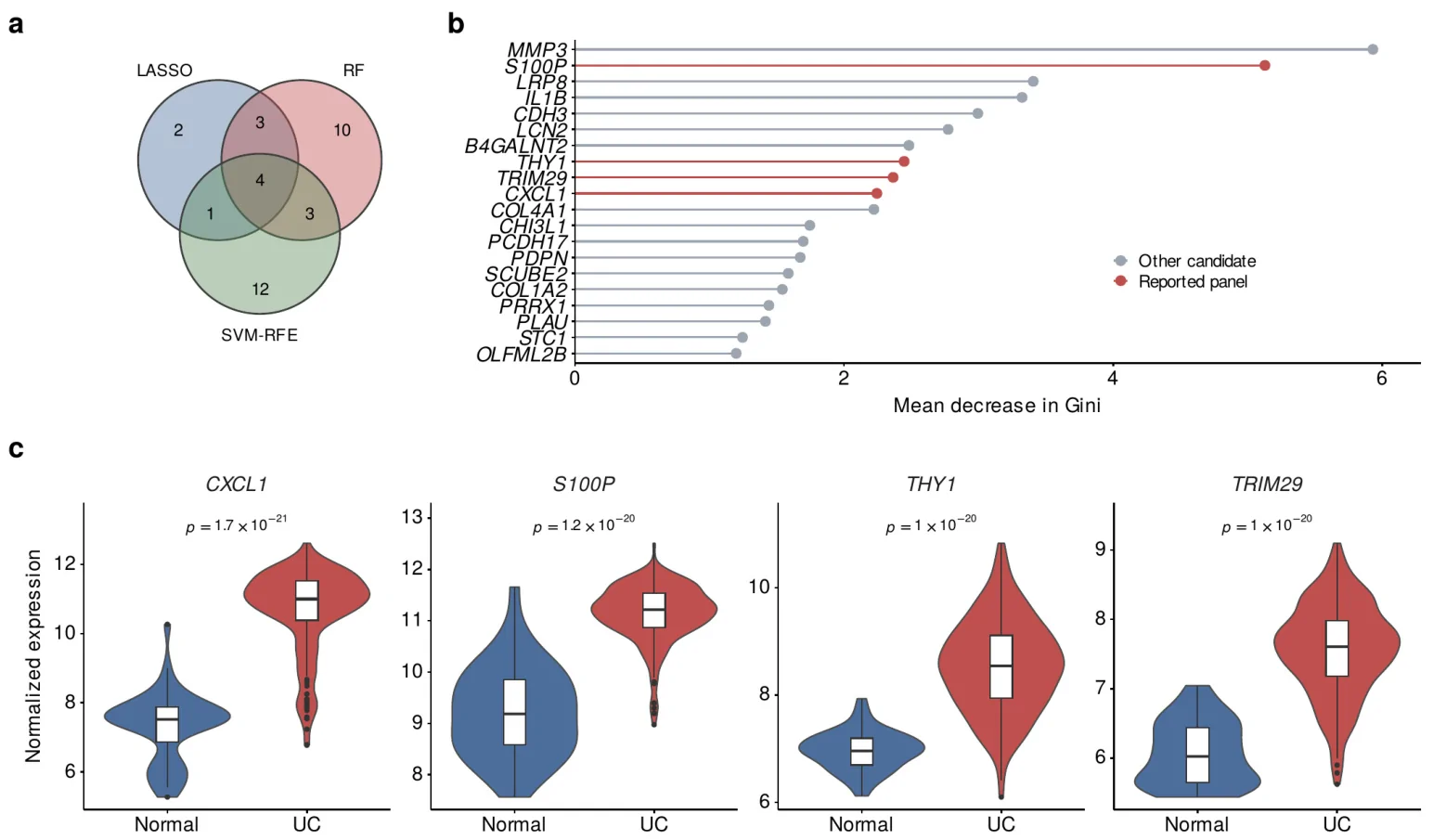
Ensemble machine-learning feature selection. (**a**) Venn diagram of genes selected by LASSO, random forest, and SVM-RFE; the intersection comprises four hub genes. (**b**) Random forest variable importance for the 20 highest-ranked candidates, with the reported panel highlighted; note that the four hub genes are not the four highest-ranked genes by this criterion. (**c**) Expression of the four hub genes in the harmonized UC cohort (Wilcoxon rank-sum test). This intersection is the output of a single fit; its reproducibility under resampling is examined in the following section and in Figure 4a.

To determine which of the 91 genes the algorithms would converge on without imposing biological priors, three methods were applied to the UC expression matrix: LASSO logistic regression, which retained 10 variables with non-zero coefficients; random forest, from which the 20 genes with the highest mean decrease in Gini were taken; and linear-kernel SVM-RFE, which retained 20 features. Four genes appeared in all three lists: *CXCL1*, *S100P*, *THY1*, and *TRIM29* (Figure 3a,b). Their annotated functions place them in three distinct compartments. *CXCL1* is a CXC chemokine that drives neutrophil recruitment. *S100P* belongs to the S100 family of calcium-binding proteins and has well-documented pro-tumorigenic functions. *THY1*, also known as *CD90*, is an established mesenchymal and fibroblast marker. *TRIM29* is a tripartite motif E3 ubiquitin ligase that acts at the intersection of NF-κB and interferon signalling. In the harmonized UC cohort, all four were expressed at significantly higher levels in inflamed mucosa than in healthy controls (Wilcoxon test, *p* < 0.001 for each; Figure 3c). The extent to which this convergence can be relied upon is a separate question, which is addressed directly in the following section.

### 3.4. Instability of the Selection Step Under Bootstrap Resampling

**Figure 4:**
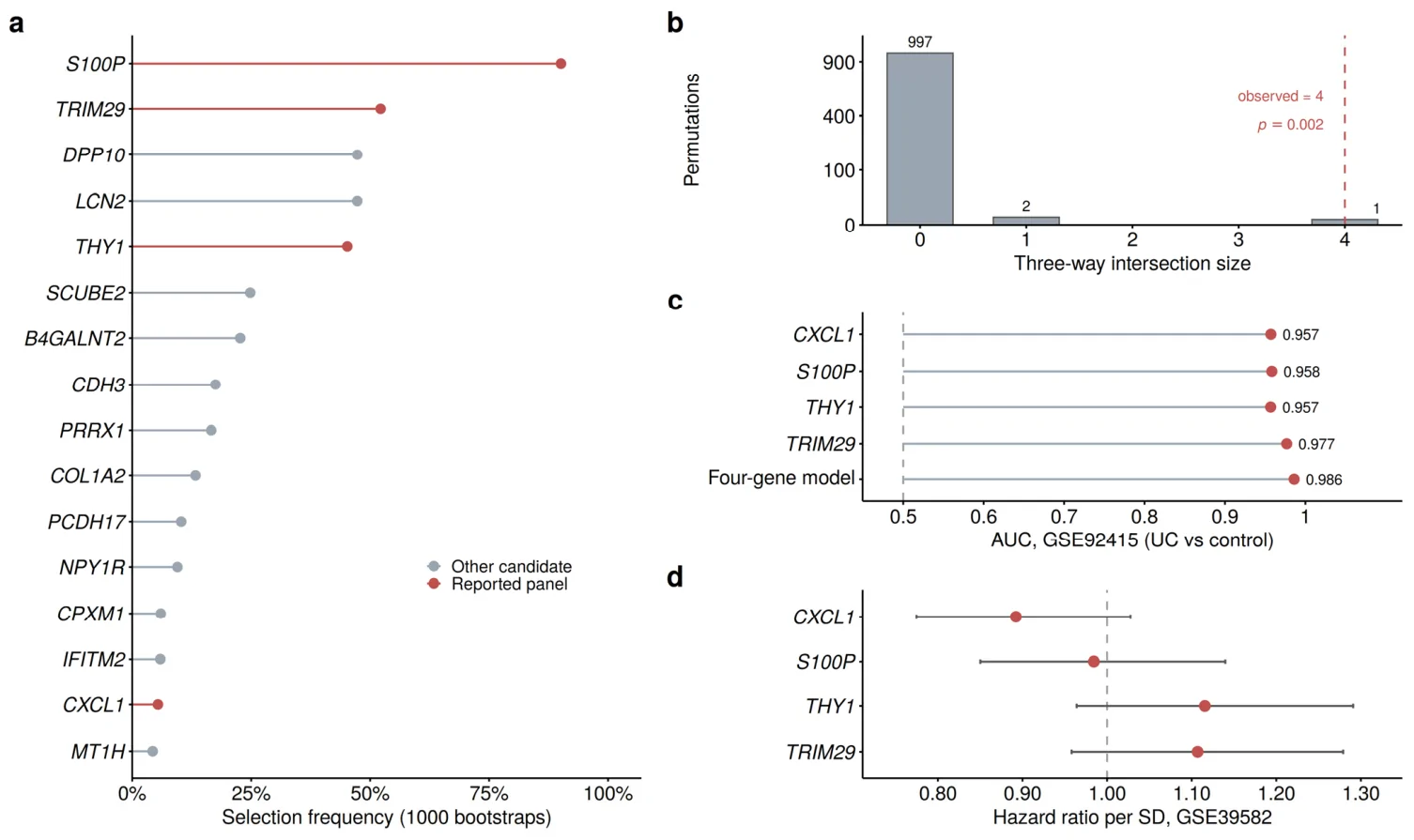
Robustness of the feature selection and external replication of the panel. (**a**) Selection frequency of the 16 most frequently selected candidate genes across 1000 bootstrap resamples of the harmonized UC cohort, defined as the proportion of resamples in which a gene was selected by LASSO, random forest, and SVM-RFE simultaneously; the four genes reported here are highlighted. (**b**) Null distribution of the three-way intersection size under 1000 permutations of disease labels, plotted on a square-root scale so that the rare non-zero counts remain visible; the observed intersection of four genes is marked. (**c**) Discrimination between inflamed and control mucosa in the independent cohort GSE92415 (162 UC, 21 control): area under the curve for each gene individually, and for a four-gene logistic model evaluated under 10 repeats of 10-fold cross-validation. (**d**) External validation of the survival association in GSE39582 (*n* = 573, 190 deaths): hazard ratio per standard deviation for each gene, with 95% confidence intervals. Red points mark the four genes of the reported panel and grey points the remaining candidates. The dashed lines mark the observed intersection size in (**b**), an area under the curve of 0.5 in (**c**), and a hazard ratio of 1 in (**d**).

A three-way intersection of feature-selection algorithms is a fragile construct, and its fragility was quantified before anything was built on it. When the full pipeline—LASSO, random forest, and SVM-RFE applied to the 91 candidates—was repeated across 1000 bootstrap resamples of the harmonized UC cohort, *S100P* was reselected in 90.1% of resamples, *TRIM29* in 52.2%, *THY1* in 45.2%, and *CXCL1* in 5.4%; all four were recovered together in 0.3%. One gene outside the panel, *DPP10*, was selected more often (47.3%) than *THY1* (Figure 4a). Omitting either source dataset also changed the result: rerunning the pipeline in GSE87466 alone recovered none of the four genes, and in GSE75214 alone recovered one (*S100P*). Part of this instability reflects collinearity rather than noise. The 91 candidates are strongly co-expressed, and, in the stromal and epithelial compartments, the genes that displace the hub genes are close substitutes for them: *THY1* correlates at *r* = 0.88–0.94 with *BGN*, *OLFML2B*, *COL4A1*, *PDPN*, and *COL5A2*, all of which are stromal markers by any definition, and *TRIM29* correlates at *r* = 0.82 and 0.73 with *CDH3* and *S100P*, respectively. When each candidate was assigned to its nearest hub gene by correlation and the frequency with which each compartment was represented was determined, the epithelial and stromal compartments were recovered in most resamples, but all three compartments together in only 10.8%; when *CXCL1* itself was not selected, another gene of the chemokine–myeloid compartment appeared in only 12.8% of resamples. The intersection is nonetheless far from a chance result: permuting disease labels 1000 times and repeating the pipeline gave a median three-way intersection of zero genes, with an intersection as large as the observed one in 1 of 1000 permutations (*p* = 0.002; Figure 4b). We interpret this as indicating that the ensemble identifies a genuine and non-random signal but does not identify a unique or reproducible set of genes within it. The four genes are therefore treated as a hypothesis-generating selection, and the sections that follow ask whether independent evidence supports them rather than relying on the selection step itself. Selection frequencies for the most frequently selected candidates are shown in Figure 4a, and the full frequencies together with compartment assignments are given in Supplementary Table S1.

### 3.5. Replication of the Panel in an Independent UC Cohort

The four genes were next tested in GSE92415, a UC mucosal cohort that did not contribute to discovery (162 UC and 21 control samples on the same array platform as GSE87466). All four were more highly expressed in UC mucosa, matching the direction observed in discovery (Wilcoxon *p* ≤ 1.3 × 10^−11^ for each), and each discriminated inflamed from control mucosa on its own, with areas under the curve of 0.957 for *CXCL1*, 0.958 for *S100P*, 0.957 for *THY1*, and 0.977 for *TRIM29* (Figure 4c). A four-gene logistic model gave an apparent AUC of 0.999 and a cross-validated AUC of 0.986 (95% CI 0.965–1.000; Figure 4c) under 10 repeats of 10-fold cross-validation; the cross-validated value is reported here because the apparent value is fitted and evaluated on the same samples (Supplementary Table S5). This replication concerns the differential expression in UC mucosa, which is the property on which the rest of the study builds; it does not by itself establish relevance to colorectal carcinogenesis.

### 3.6. Absence of Evidence for an Upstream Germline Effect in Mendelian Randomization

**Figure 5:**
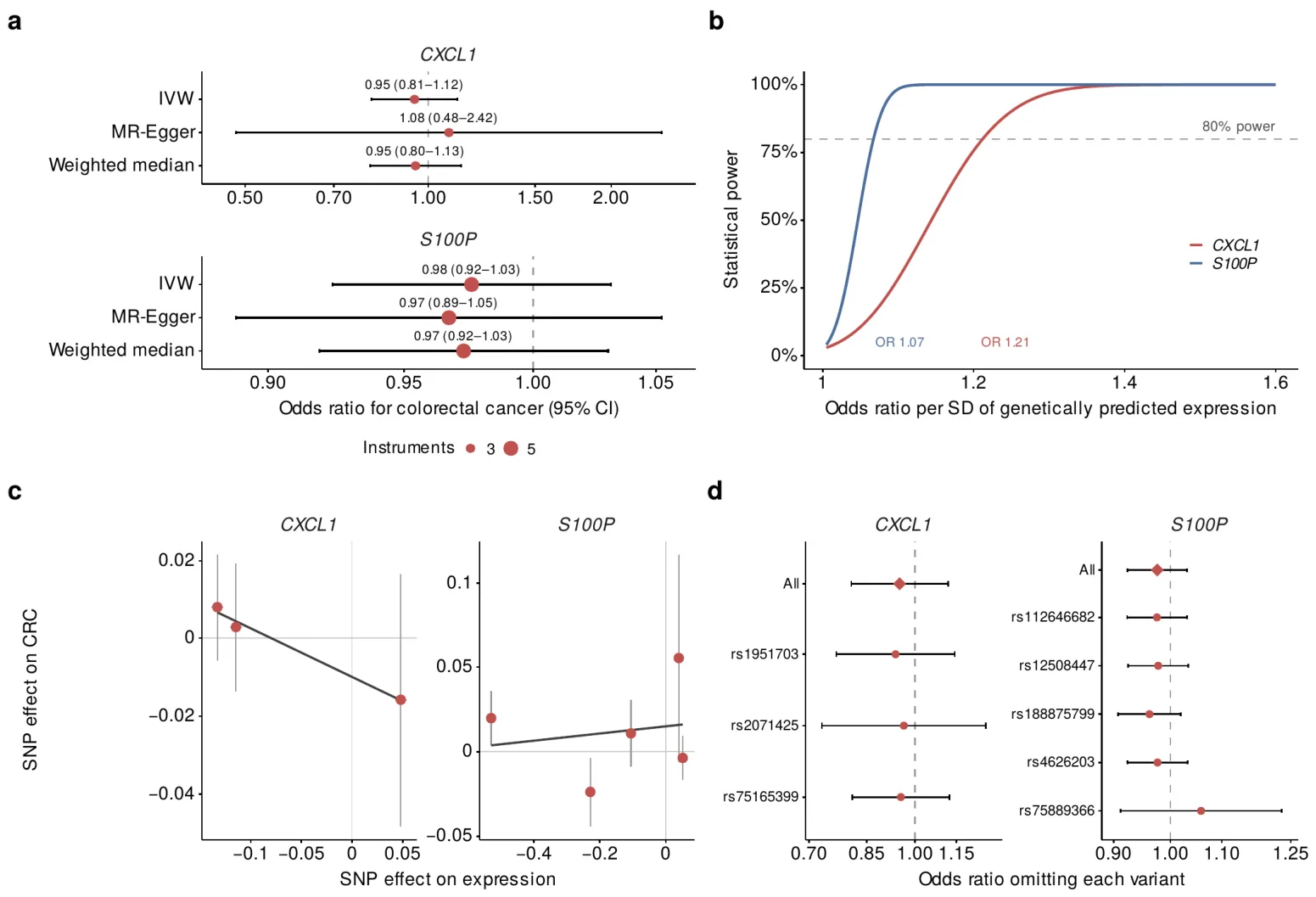
Two-sample Mendelian randomization of hub gene expression on colorectal cancer risk. (**a**) Forest plot of the inverse-variance weighted, MR-Egger, and weighted median estimates for *CXCL1* and *S100P*; point size is proportional to the number of instruments and all confidence intervals cross the null. (**b**) Statistical power of the present design as a function of the true odds ratio per standard deviation of genetically predicted expression, given the instrument strength actually achieved; the dashed line marks 80% power and the annotated values are the smallest odds ratios detectable at that threshold, 1.07 for *S100P* and 1.21 for *CXCL1*. (**c**) SNP effect on expression against SNP effect on colorectal cancer, with the inverse-variance weighted slope. (**d**) Leave-one-out analysis; the diamond marks the estimate obtained using all instruments. Red points are the individual instruments in (**c**) and the leave-one-out estimates in (**d**); horizontal bars are 95% confidence intervals and the grey whiskers in (**c**) are the standard errors of the two SNP effects. The dashed vertical lines in (**a**,**d**) mark an odds ratio of 1. *THY1* and *TRIM29* do not appear because eQTLGen contains no cis-eQTL for either gene at FDR < 0.05.

Having identified the panel, we next asked whether the germline variation in the expression of these genes affects the CRC risk. A two-sample MR analysis was performed, taking cis-eQTLs as instruments and a large CRC GWAS as the outcome. Three instruments survived clumping for *CXCL1* and five for *S100P*, and both returned null estimates. Under the inverse-variance weighted (IVW) estimator, *CXCL1* gave an odds ratio of 0.95 (95% CI 0.81–1.12, *p* = 0.533) and *S100P* gave 0.98 (95% CI 0.92–1.03, *p* = 0.386); MR-Egger and the weighted median agreed (Table 1, Figure 5a). Unlike many eQTL-based MR analyses, this one is not limited by weak instruments. The three *CXCL1* variants explain 3.3% of the expression variance (per-SNP *F* = 64–546; overall *F* = 345) and the five *S100P* variants explain 28.4% (per-SNP *F* = 36–8674; overall *F* = 2423). Against 6581 cases and 463,421 controls, this gives 80% power to detect an odds ratio of 1.21 or beyond for *CXCL1* and 1.07 or beyond for *S100P* per standard deviation of genetically predicted expression (Figure 5b; Supplementary Table S2). The *S100P* result is therefore an informative null: an effect of the size usually reported for a bona fide risk gene would have been detected. For *CXCL1*, the null excludes moderate-to-large effects but leaves smaller ones open. There was no evidence of directional horizontal pleiotropy (MR-Egger intercept *p* = 0.80 for both; Figure 5c), Cochran’s Q gave no evidence of heterogeneity (*CXCL1 Q* = 0.21, *p* = 0.900; *S100P Q* = 3.32, *p* = 0.506), leave-one-out analysis showed that neither estimate depended on a single variant (Figure 5d), funnel plots showed no asymmetry (Supplementary Figure S3), and MR-Steiger confirmed that the instruments explain far more variance in expression than in CRC risk (*p* = 1.9 × 10^−198^ and *p* < 10^−300^), confirming that the direction of the test is correct. *THY1* and *TRIM29* could not be tested at all, and the reason is more absolute than a clumping threshold: neither gene has a single cis-eQTL in eQTLGen at FDR < 0.05, so no instrument exists at any significance threshold (Supplementary Table S2). Taken together, the germline-determined blood expression of *CXCL1* and *S100P* shows no detectable effect on CRC risk. We emphasize what this does not establish: MR tests the lifelong genetically determined expression, not the inflammation-induced expression these genes show in diseased mucosa, and a null result here is not positive evidence that they act as downstream mediators. This point is taken up in the Discussion section.

IVW, inverse-variance weighted; OR, odds ratio; CI, confidence interval. Instruments are cis-eQTLs from the eQTLGen consortium (whole blood, *n* = 31,684); the outcome is colorectal cancer (IEU OpenGWAS accession ebi-a-GCST90018808; 6581 cases and 463,421 controls). MR-Egger intercept *p* = 0.80 for both exposures. *THY1* and *TRIM29* have no cis-eQTL at FDR < 0.05 in eQTLGen and are therefore untestable rather than merely under-instrumented. Instrument F statistics, variance explained, power estimates, and MR-Steiger results are given in Supplementary Table S2.

### 3.7. Association of Hub Genes with Immune Compartments and Checkpoint Expression

**Figure 6:**
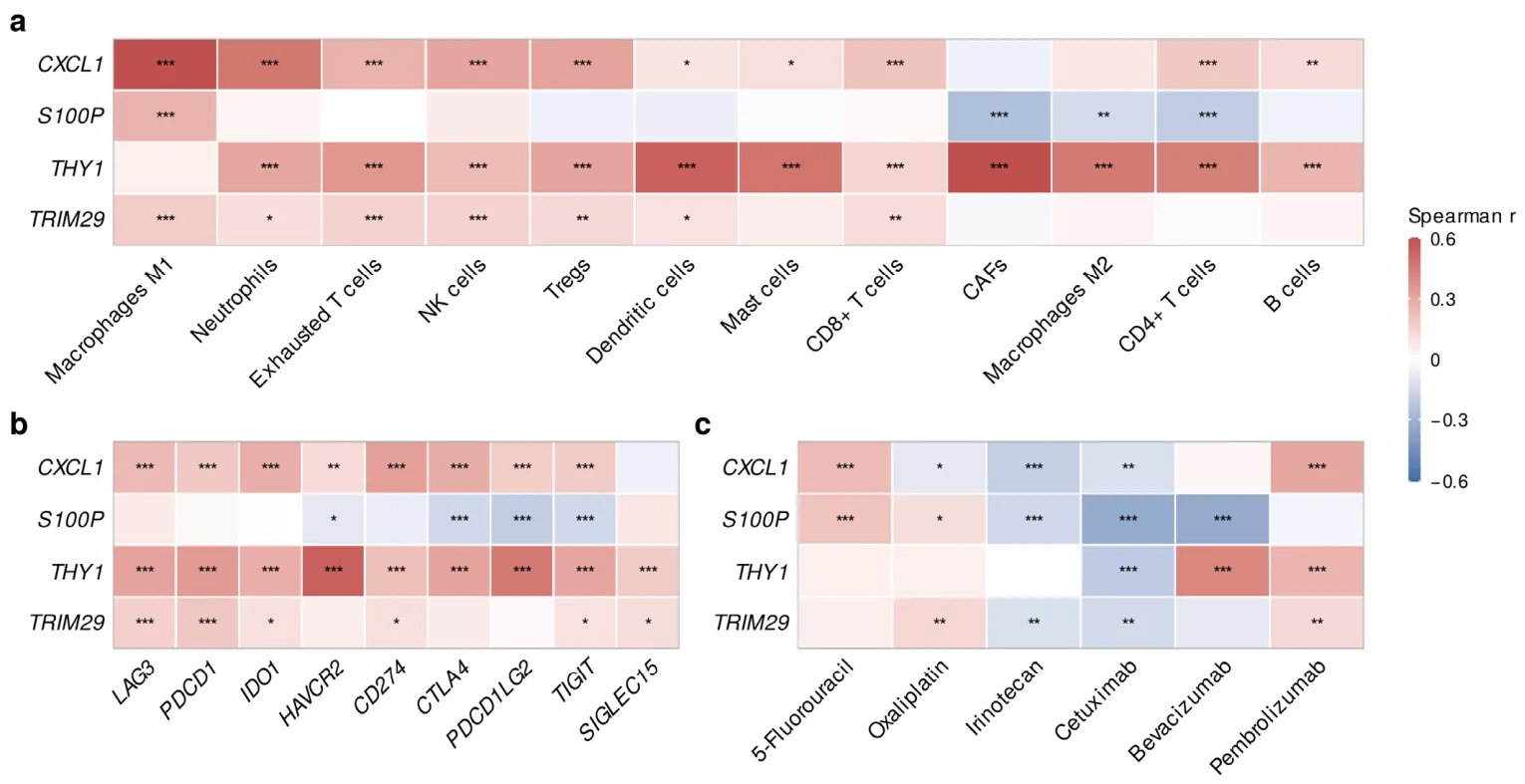
Associations of hub genes with the TCGA-COAD immune microenvironment and therapeutic pathway signatures. (**a**) Spearman correlations between hub gene expression and ssGSEA scores for 12 immune cell types. (**b**) Correlations with nine immune checkpoint genes. (**c**) Correlations with pathway signature scores for six colorectal cancer therapies. The three panels share a single colour scale, truncated at ±0.6 so that the few coefficients beyond that range are drawn at the limiting colour. Asterisks denote * *p* < 0.05, ** *p* < 0.01, and *** *p* < 0.001. The signature scores in (**c**) describe transcriptional pathway activity computed from expression data; no clinical treatment-response information was available for this cohort and these correlations should not be read as evidence of differential drug response.

The relationship between the four genes and the CRC immune landscape was examined by applying ssGSEA to estimate the abundance of 12 immune cell types in TCGA-COAD tumours. *CXCL1* showed the strongest and most distinctive immune footprint: it was positively correlated with M1 macrophages and neutrophils (*r* = 0.66, *p* < 0.001), exhausted T cells (*r* = 0.51), and regulatory T cells (*r* = 0.48), whereas its associations with CAFs and M2 macrophages did not reach significance. *THY1* showed a different pattern, tracking most closely with CAFs (*r* > 0.5), dendritic cells, and M2 macrophages, which is consistent with its identity as a mesenchymal marker. *S100P* was correlated in the opposite direction for several of these compartments, being negatively correlated with CAFs, M2 macrophages, and CD4^+^ T cells. *TRIM29* showed broad but modest positive correlations across all compartments (Figure 6a). Among immune checkpoint molecules, all four genes were significantly correlated with multiple checkpoints (Figure 6b). *THY1* was the most consistent of the four, being positively associated with all nine checkpoints examined, mostly in the range *r* = 0.3–0.5. We also examined whether the genes track with the transcriptional pathway signature scores for six standard CRC therapies, and found differentiated association patterns (Figure 6c; Supplementary Figure S4). These are correlations with pathway activity scores computed from expression data; no treatment-response information was available in this cohort, and they should not be read as evidence of differential drug response.

### 3.8. Retention of CXCL1 Alone by LASSO-Cox and Failure of the Prognostic Signal to Replicate

**Figure 7:**
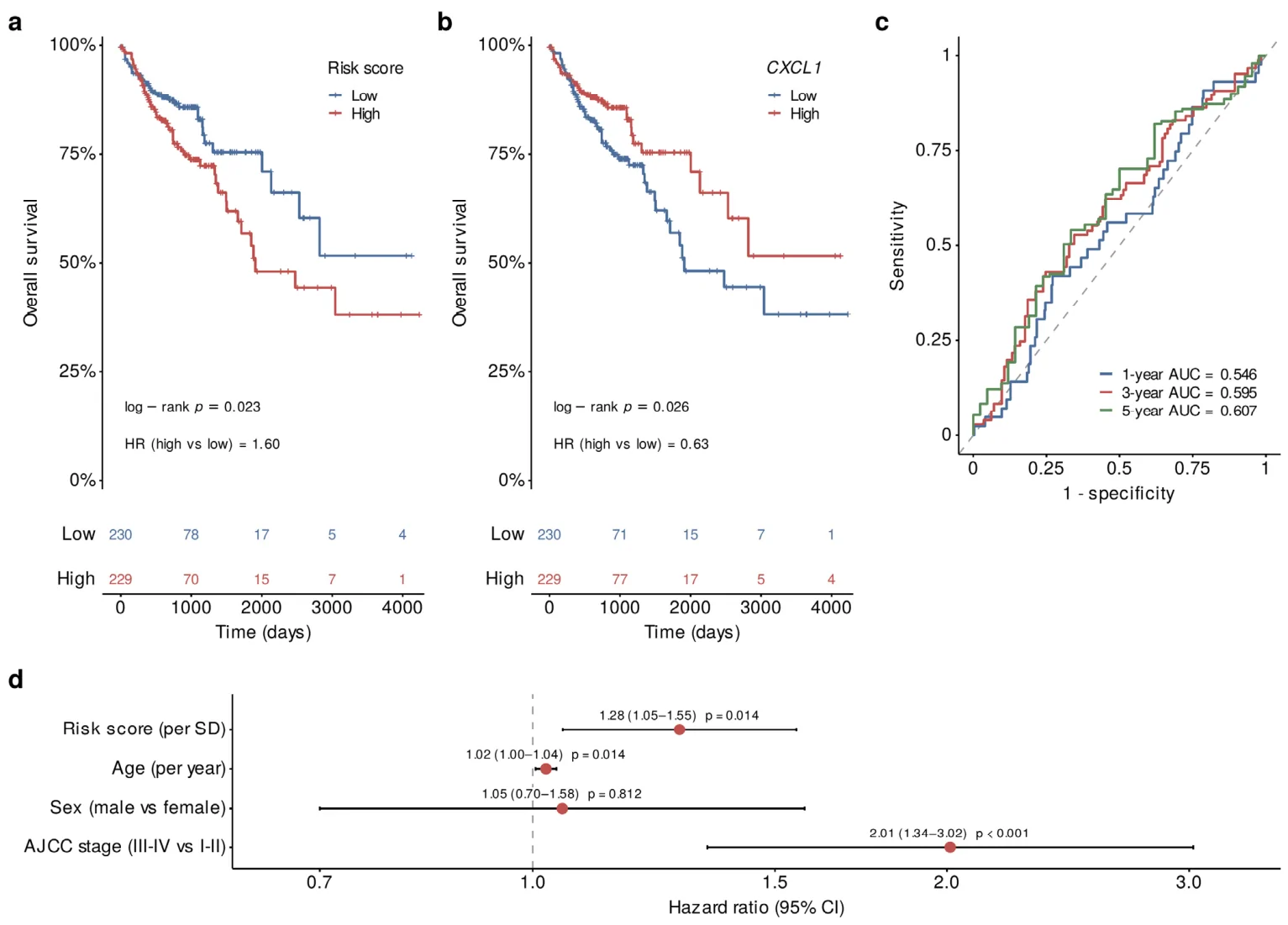
Survival analysis of the *CXCL1*-based risk score in TCGA-COAD (*n* = 459, 97 deaths). LASSO-Cox regression over the four hub genes retained a single non-zero coefficient, for *CXCL1* (β = −0.0693), so the risk score is a linear function of that gene alone. (**a**) Kaplan–Meier curves stratified at the median risk score, with numbers at risk below. (**b**) Kaplan–Meier curves stratified at median *CXCL1* expression; higher expression is associated with better rather than worse overall survival. (**c**) Time-dependent ROC curves at 1, 3, and 5 years. (**d**) Multivariable Cox model adjusted for age, sex, and AJCC stage, with the risk score expressed per standard deviation. In (**a**,**b**), blue denotes the low group and red the high group, and the numbers at risk beneath each curve are coloured to match; the dashed vertical line in (**d**) marks a hazard ratio of 1. Optimism-corrected discrimination (bootstrap-corrected C-index 0.626 for the full model and 0.556 for the score alone) and the external validation in GSE39582, which did not reach significance, are reported in the text; the nomogram and calibration curves are given in Supplementary Figure S5. This analysis is exploratory and the score is not a validated prognostic model. In (**a**,**b**) the table of numbers at risk printed beneath the curves belongs to the panel directly above it and is not a separate panel. The diagonal dashed line in (**c**) marks an area under the curve of 0.5, the line of no discrimination.

We next asked whether the panel carried prognostic information in TCGA-COAD (*n* = 459 patients with survival annotation, 97 deaths). LASSO-Cox regression applied to the four genes retained a single non-zero coefficient, for *CXCL1* (β = −0.0693); the coefficients for *S100P*, *THY1*, and *TRIM29* were shrunk to zero. The resulting risk score is therefore a linear function of *CXCL1* alone and correlates with it at *r* = −1.00, and it is described as such rather than as a four-gene score. The sign of the coefficient deserves attention: because it is negative, a higher *CXCL1* expression corresponds to a lower risk score, and, in univariable analysis, a higher *CXCL1* was associated with better overall survival (HR 0.79 per SD, 95% CI 0.65–0.96, *p* = 0.020). None of the other three genes reached significance (*S100P* HR 0.89, *p* = 0.214; *THY1* HR 1.09, *p* = 0.378; and *TRIM29* HR 0.99, *p* = 0.950). Splitting the cohort at the median score separated the two groups in a Kaplan–Meier analysis (log-rank *p* = 0.023; Figure 7a,b), and, after adjustment for age, sex, and AJCC stage, the score remained significant on a standardized scale (HR 1.28 per SD, 95% CI 1.05–1.55, *p* = 0.014; Figure 7d, Table 2). Time-dependent AUCs were 0.55, 0.60, and 0.61 at 1, 3, and 5 years (Figure 7c). Internal validation makes the limits of the model plain: it reached an apparent C-index of 0.642, falling to 0.626 after optimism correction with 1000 bootstrap resamples and 0.631 under repeated 10-fold cross-validation, and most of that discrimination derives from age and stage, the score by itself giving a corrected C-index of 0.556. The proportional-hazards assumption held for the score (Schoenfeld *p* = 0.40); the significant global test (*p* = 0.033) is attributable to the AJCC stage (*p* = 0.009). A nomogram fitted on the complete-case subset (*n* = 279, 54 deaths; Supplementary Figure S5b) was calibrated reasonably at 1 year and drifted at 3 and 5 years (Supplementary Figure S5c). Most importantly, the signal did not replicate: in GSE39582 (*n* = 573, 190 deaths), the same *CXCL1*-based score gave an HR of 1.12 per SD (95% CI 0.97–1.29, *p* = 0.114) with a C-index of 0.54 (Figure 4d), and none of the four genes individually reached significance, although the direction of the *CXCL1* effect matched that in TCGA-COAD (HR 0.89 per SD, 95% CI 0.78–1.03; Supplementary Table S5). We therefore make no prognostic claim for this panel. What the survival analysis contributes is a caution about the mechanism, discussed below: in bulk CRC transcriptomes, a high *CXCL1* marks tumours with a better rather than a worse outcome.

HR, hazard ratio; CI, confidence interval. The risk score is a linear function of *CXCL1* alone (β = −0.0693), the only gene retained by LASSO-Cox. Because the per-unit scale of the score is arbitrary, the standardized (per standard deviation) estimate is the interpretable one and is the estimate quoted in the text. The model was refitted during revision with age in years and AJCC stage dichotomized as I–II versus III–IV. Optimism-corrected discrimination is reported in the text; the score is exploratory and is not a validated prognostic model.

### 3.9. Reproducible Compartment Structure but Unresolved Group Differences in Spatial Mapping

**Figure 8:**
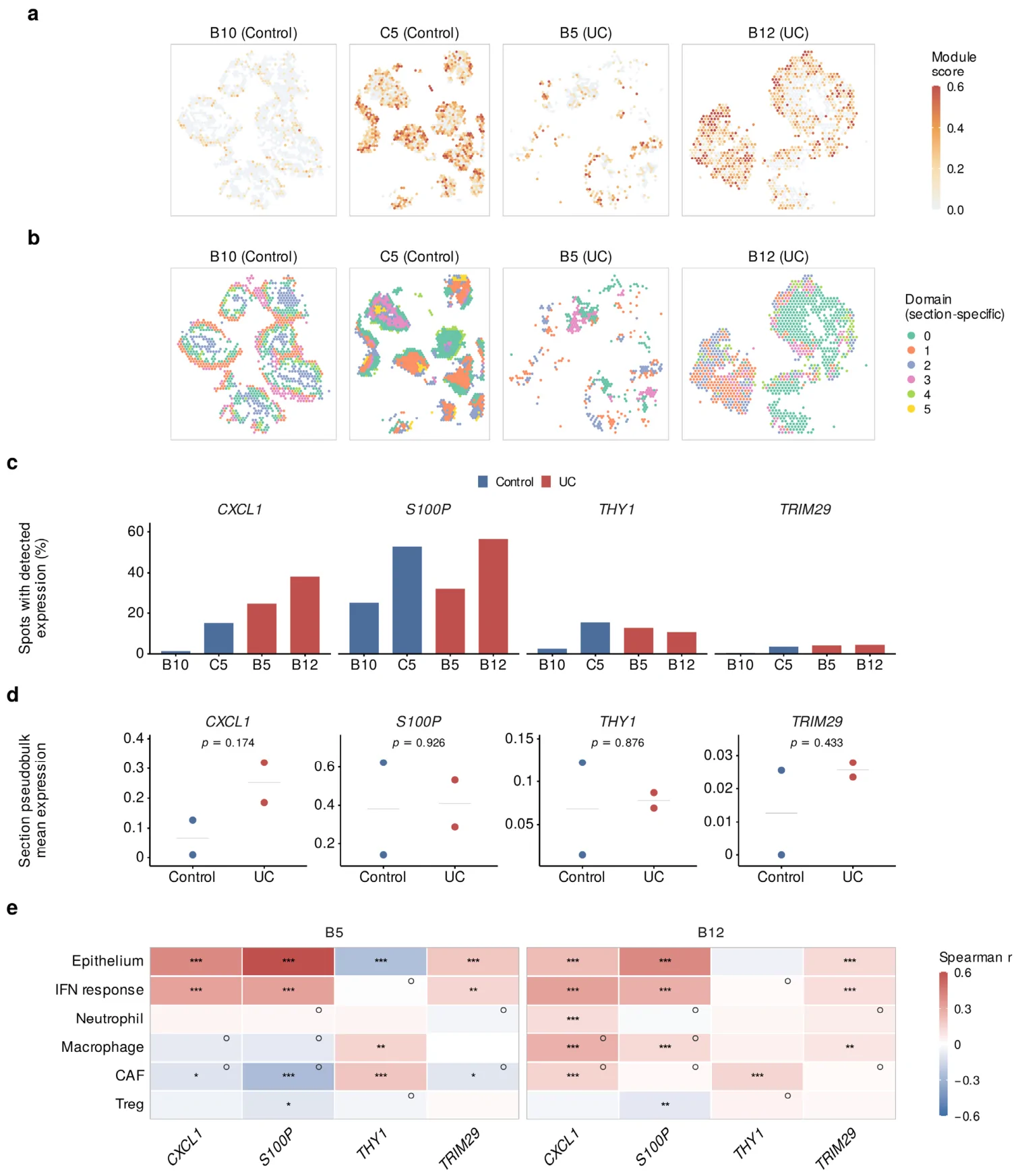
Exploratory Visium spatial mapping in UC mucosa. (**a**) Spatial distribution of the four-gene module score across two control (B10, C5) and two UC (B5, B12) sections; grey spots carry a score of zero and the colour scale is truncated at 0.6, so the few spots above that value are drawn at the limiting colour. (**b**) Unsupervised spatial clustering. Domains were called independently within each section, so the numbering is section-specific and a given domain number does not denote the same tissue compartment across sections; inflammatory niches were present in both UC sections and in neither control. (**c**) Proportion of spots with detected expression of each hub gene, by section. (**d**) Section-level pseudobulk mean expression (points, individual sections; horizontal bar, and group mean), annotated with *p* values from a linear mixed model fitted to the spot-level data with section as a random intercept. Spots within a section are not independent biological replicates, and with two sections per group, no gene reaches significance; the control section C5 exceeds the other control section B10 for all four genes, which is the principal reason the section-level comparison is null. (**e**) Spearman correlations between hub gene expression and cell-type module scores, computed separately within each UC section, on a colour scale truncated at ±0.6; asterisks follow the convention given in Figure 6. A circle in the upper-right corner of a cell marks the 9 of 24 gene–compartment pairs whose correlation changes sign between the two sections; all of these are weak in both sections (|*r*| < 0.27), and every pair on which the three-compartment model depends is consistent.

All preceding analyses were conducted at the bulk-tissue level, which cannot resolve where within the tissue a gene is expressed. Visium spatial transcriptomic data were therefore analysed from four colonic biopsies, two from UC patients (B5 and B12) and two from healthy controls (B10 and C5). One constraint should be stated before any result is presented: with two sections per group, the effective sample size for a UC-versus-control comparison is four, and the several thousand spots those sections contain are not independent biological replicates. The section was therefore treated as the unit of replication. After quality filtering (450–1240 spots per section) and SCTransform normalization, the four-gene module score and the highest-scoring individual spots fell in regions that appeared inflamed on histology (Figure 8a), and unsupervised clustering identified four to six transcriptomic domains per section, with inflammatory niches present in both UC sections and in neither control (Figure 8b). The quantitative comparison, however, does not support a group difference. At the section level, only *CXCL1* was higher in both UC sections than in either control (pseudobulk means 0.185 and 0.320 in UC versus 0.009 and 0.127 in control; Figure 8d), and the proportion of spots with a detected expression follows the same ordering (Figure 8c); for *S100P*, the highest value of the four sections was in a control (C5, 0.624), and *THY1* and *TRIM29* showed no separation. Fitting the spot-level data as a linear mixed model with the section as a random intercept returns no significant difference for any of the four genes (*CXCL1* β = 0.19, *p* = 0.174; *S100P p* = 0.926; *THY1 p* = 0.876; *TRIM29 p* = 0.433; Supplementary Table S3), which is what the four sections can support. Spot-level distributions for each section are shown in Supplementary Figure S7. The contrast with the naive spot-level test is instructive and worth reporting: treating each spot as an independent observation gives *p* = 5.7 × 10^−67^ for *CXCL1* and *p* = 1.5 × 10^−5^ for *S100P* (Supplementary Table S3). These values are artefacts of pseudoreplication; an earlier version of this manuscript quoted them as evidence, and we no longer do so.

What the spatial data do support is the compartment assignment, and, here, the evidence is internally consistent. Merging the two UC sections (1516 spots) and correlating the hub gene expression with cell-type module scores, *CXCL1* tracked with the epithelium (*r* = 0.30) and interferon-response signal (*r* = 0.33), with weaker associations to macrophages (*r* = 0.22) and neutrophils (*r* = 0.12); *THY1* was the most stroma-restricted of the four, correlating with CAFs (*r* = 0.14) and negatively with the epithelial signal (*r* = −0.11); *S100P* showed the strongest compartment preference of all, correlating with the epithelium at *r* = 0.50 and with the interferon response at *r* = 0.35; and *TRIM29* occupied a similar epithelial–interferon position (r ≈ 0.12; merged-pool coefficients, shown per section in Figure 8e). Because *p* values computed across 1516 non-independent spots are governed by the spot count, these coefficients are assessed instead by their reproducibility between the two UC sections. In total, 15 of the 24 gene-compartment pairs kept the same sign in both sections, and every pair on which the three-compartment model depends was among them: *S100P*–epithelium (*r* = 0.62 and 0.41), *THY1*–CAF (0.20 and 0.14), *THY1*–epithelium (−0.26 and −0.05), *CXCL1*–epithelium (0.41 and 0.23), *CXCL1*–interferon (0.32 and 0.32), and *TRIM29*–epithelium (0.20 and 0.13) (Supplementary Table S4). The nine pairs that changed sign were weak in both sections (|*r*| < 0.27) and none is load-bearing. Apart from the *S100P*–epithelium pair, the coefficients lie mostly between 0.1 and 0.35; these are modest effects, and we claim not one-to-one co-localization but only a directional spatial preference that is consistent across the two sections available.

## 4. Discussion

Rather than claiming novelty for each individual gene, we emphasize that these four markers occupy three spatially distinguishable compartments, and that the assignment is reproducible between the two UC sections we examined. Two qualifications frame everything that follows. First, all of the evidence here is computational and drawn from public cohorts; no compartment assignment has been confirmed at the protein level in tissue. Second, the ensemble feature selection that nominated these four genes is not itself reproducible under resampling, which means the panel should be read as one representative set drawn from a larger co-regulated programme rather than as an optimal or unique solution. What supports the panel is not the selection step but what the four genes do afterwards: they are strongly and consistently differentially expressed in an independent UC cohort, they occupy consistent tissue compartments, and each carries a distinct immune-correlation profile.

On the immune axis, *CXCL1* has the most clearly defined mechanistic role of the four genes. It is consistently overexpressed in CRC relative to normal mucosa, and there is good evidence that an elevated *CXCL1* can exacerbate colitis [18], consistent with a broader framework in which inflammatory mediators such as prostaglandins contribute to CRC progression [19]. The position of *CXCL1* within the signalling cascade is instructive. Tumour cells, CAFs, endothelial cells, and myeloid populations in the CRC microenvironment all release CXC chemokines, *CXCL1* among them. These establish local concentration gradients that recruit granulocytic-myeloid-derived suppressor cells and tumour-associated neutrophils through CXCR2. Once recruited, these neutrophils express high levels of PD-L1 and actively suppress CD8^+^ T cells, contributing to an immunosuppressive microenvironment that favours tumour progression. The canonical pathway here is the *CXCL1*/8-CXCR2 axis downstream of *SMAD4* loss [20], and a parallel route operates through IL-23/IL-17 to drive colonic tumour growth [21]. The correlations observed here, with neutrophils and exhausted T cells, are consistent with this ‘inflammation → *CXCL1* → myeloid recruitment → T cell exhaustion’ cascade. The survival data, however, do not fit this model neatly, and the discrepancy is worth stating rather than reconciling superficially: in TCGA-COAD, a higher *CXCL1* was associated with a better overall survival (HR 0.79 per SD, *p* = 0.020), with the same direction of effect in GSE39582. A plausible reconciliation is that, in bulk tumour transcriptomes, *CXCL1* is primarily a marker of an inflamed, immune-infiltrated tumour, and immune infiltration is one of the more reliable favourable prognostic features in CRC; the pro-tumour arm of the CXCR2 axis may be real but is not what a bulk chemokine measurement captures. Distinguishing these possibilities requires the cell-resolved measurement of which cells produce *CXCL1* and which are recruited in response, rather than further bulk correlation.

On the stromal axis, *THY1* (*CD90*) has an established identity in the CRC literature. It serves as a pan-CAF marker, and Thy-1^+^ CAFs that co-express α-SMA and *FAP* tend to cluster at the invasive front and around blood vessels, where their presence has been linked to worse clinical outcomes. These CAFs are a major local source of interleukin-6 and related soluble mediators, through which they reshape the surrounding immune compartment and support tumour-cell stemness and invasion [22]; in colorectal tumours specifically, CD90+ stromal cells have been identified as the dominant local source of interleukin-6 and shown to sustain both stem-like tumour cells and mucosal inflammation [23], which is the same marker that identifies the stromal compartment described here. CAFs activated during chemotherapy have likewise been reported to sustain colorectal cancer-initiating cells and drug resistance through paracrine interleukin-17A signalling [24]. Our data are consistent with this picture: *THY1* correlated with CAF and macrophage signatures in the bulk analysis, and, in the spatial data, it lay within stromal regions that were anticorrelated with the epithelial signal.

On the epithelial axis, *S100P* and *TRIM29* shift attention to the cellular compartment in which transformation originates. *S100P* is a calcium-binding protein of the S100 family and a known downstream target of *MACC1*, a metastasis driver. It promotes CRC migration, invasion, and liver metastasis, and has been proposed as a prognostic marker for metastasis-free survival [25]. The broader S100 family is closely involved in inflammation-to-cancer biology: S100A8 and S100A9, for example, bind RAGE and TLR4 and thereby activate STAT3 and NF-κB [26]. This family-level evidence supports reading *S100P* as a marker of epithelial transformation arising on an inflammatory background [27]. *TRIM29* is the least well-characterized member of the panel. As an E3 ubiquitin ligase of the tripartite motif family, it modulates interferon signalling and NF-κB through TBK1 and STING, with more recent work suggesting roles in barrier maintenance and immune evasion [28]. We have positioned it at an epithelial–interferon node, although this assignment remains provisional and will require dedicated experimental validation.

The MR analysis requires careful reading, because a null of this kind is easily over-interpreted in either direction. For both genes that could be tested, the estimates were null and, unusually for eQTL-based MR, the instruments were strong enough for that to be informative. The five *S100P* variants explain 28.4% of the expression variance, giving 80% power to detect an odds ratio of 1.07 per standard deviation; an effect of the magnitude usually reported for an established risk gene would not have been missed. For *CXCL1*, with 3.3% of the variance explained, the corresponding threshold is 1.21, so moderate-to-large effects are excluded and smaller ones are not. *THY1* and *TRIM29* have no cis-eQTL in eQTLGen at FDR < 0.05 and cannot be tested at all with the currently available data. Two boundaries limit how far this generalizes. The instruments derive from whole blood rather than colonic mucosa, and cis-eQTL effects are frequently tissue-specific. More fundamentally, MR interrogates the lifelong genetically determined expression, whereas the biology at issue is the expression induced by chronic inflammation in the mucosa; a gene may be an important intermediate in the second sense while showing no effect in the first. An earlier version of this manuscript read the null estimates as positive evidence that these genes are inflammation-driven mediators. That inference does not follow and has been withdrawn. What can be stated is narrower but still useful: the germline-determined expression of *CXCL1* and *S100P* does not detectably influence the CRC risk, so neither is likely to be a target for genetically informed risk prediction, and whether they function as mediators of the inflammation-to-cancer transition remains an open question that would require colon tissue eQTLs, formal mediation analysis, or experimental perturbation to settle. This is broadly consistent with earlier MR work reporting that genetic-level causality between inflammatory bowel disease and CRC is difficult to establish [29]. Yu et al. recently used a similar UC-CRC shared-gene strategy supported by MR to identify *MMP1* as a protective diagnostic marker [10]; what distinguishes the present study is not the MR result but the spatial resolution of the panel across three compartments.

The spatial data provide morphological context for the three-compartment model, within the limits of a four-section dataset. The negative correlation between the *THY1* and epithelial signal mirrors the histology of UC, in which fibrotic, stroma-rich patches are spatially separated from epithelial crypts; in CRC, this separation erodes further as CAFs infiltrate the tumour parenchyma. At the other extreme, the *S100P* and epithelium are tightly co-localized, reinforcing the interpretation that *S100P* is genuinely epithelial in origin. *CXCL1* occupies an intermediate position, co-localizing with both immune and epithelial signatures, as would be expected for a chemokine produced by an inflamed epithelium that subsequently recruits immune cells. The reproducibility of these assignments between two independent sections is the strongest form of support the dataset can offer, and it should be distinguished from what the dataset cannot offer. Four sections cannot establish that the module expression differs between the UC and control mucosa; the mixed-model analysis returns no significant difference for any of the four genes, and, for *S100P*, the highest section-level value was in a control biopsy. That the same comparison yields *p* values in the range of 10^−67^ when spots are treated as independent illustrates how misleading spot-level inference can be, and we encourage others working with small Visium datasets to report the section-level analysis alongside it.

Several caveats should be acknowledged, and the analyses added during revision have sharpened rather than softened them. First, the ensemble feature selection is not reproducible: across 1000 bootstrap resamples of the discovery cohort, *S100P* was reselected in 90% of resamples, *TRIM29* in 52%, *THY1* in 45%, and *CXCL1* in only 5%, and all four were recovered together in 0.3%. Omitting either source dataset recovered none of the four genes from GSE87466 and one from GSE75214. Some of this reflects the collinearity within a co-regulated programme, in that *THY1* is frequently replaced by *BGN*, *COL1A2*, or *PRRX1* (*r* = 0.88–0.94), which are stromal markers by any account, but not all of it: when *CXCL1* was not selected, another gene of the chemokine–myeloid compartment appeared in only 13% of resamples. The size of the three-way intersection is nonetheless far from random (the median intersection of 0 under 1000 label permutations, *p* = 0.002). The conclusion we draw is that the intersection identifies a real signal while the identity of the genes within it is unstable, and that the panel should be treated accordingly. Second, the MR analysis relies on blood cis-eQTLs and, for two of the four genes, on instruments that do not exist. Third, the survival analysis produced a single-gene score whose discrimination is weak, whose external replication failed to reach significance, and whose direction of effect complicates rather than supports the mechanistic narrative. Fourth, the spatial dataset comprises four sections with no dysplasia or early colitis-associated cancer, so the module cannot be read as a trajectory. Fifth, the drug-pathway correlations are associations with transcriptional signature scores rather than measurements of clinical response. Sixth, discovery was performed in bulk transcriptomes, so regulators confined to rare cell populations would not have been detected. Finally, no in vivo or patient-tissue validation has been performed. Moving the compartment assignments from correlational to histological would require immunohistochemistry and multiplex immunofluorescence across the disease spectrum, for which *THY1*/α-SMA and *S100P*/EpCAM are obvious starting pairs, followed by functional work such as knockdown in the AOM-DSS model and in CAF–epithelium co-culture to establish directionality.

In summary, *CXCL1*, *S100P*, *THY1*, and *TRIM29* are co-regulated across UC and CRC and separated into epithelial, stromal, and immune compartments in spatial data, with the compartment assignment reproducible between independent UC sections and the differential expression reproducible in an independent UC cohort. Around that core, several of our initial expectations did not survive testing: the feature-selection convergence is not reproducible, the prognostic score reduces to *CXCL1* and does not replicate externally, the direction of the *CXCL1* survival association runs opposite to the simplest mechanistic reading, and four spatial sections cannot demonstrate a group difference. These outcomes are reported because they define what the panel can and cannot be used for. It is not a prognostic tool and, on present evidence, not a target set for chemoprevention. Its value lies in being a compartment-resolved description of the inflamed mucosa: a framework in which epithelial (*S100P* and *TRIM29*), stromal (*THY1*), and chemokine (*CXCL1*) programmes can be measured separately in the same tissue section, and a well-defined starting point for the histological and functional work that would be needed to test whether that structure carries the causal weight we have been careful not to assign it here.

## Figures and Tables

**Table 1:** Two-sample Mendelian randomization of hub gene expression on colorectal cancer risk.

Exposure	Instruments (*n*)	Method	OR (95% CI)	*p*
*CXCL1*	3	IVW	0.95 (0.81–1.12)	0.533
*CXCL1*	3	MR-Egger	1.08 (0.48–2.42)	0.880
*CXCL1*	3	Weighted median	0.95 (0.80–1.13)	0.584
*S100P*	5	IVW	0.98 (0.92–1.03)	0.386
*S100P*	5	MR-Egger	0.97 (0.89–1.05)	0.495
*S100P*	5	Weighted median	0.97 (0.92–1.03)	0.345
*THY1*	0	—	No valid instrument	—
*TRIM29*	0	—	No valid instrument	—

**Table 2:** Multivariable Cox regression for overall survival in TCGA-COAD (*n* = 459, 97 deaths).

Variable	HR (95% CI)	*p*
*CXCL1*-based risk score (per unit)	11.45 (1.64–79.76)	0.014
*CXCL1*-based risk score (per SD)	1.28 (1.05–1.55)	0.014
Age (per year)	1.02 (1.00–1.04)	0.014
Sex (male versus female)	1.05 (0.70–1.58)	0.812
AJCC stage (III–IV versus I–II)	2.01 (1.34–3.02)	<0.001

## Data Availability

All datasets analysed during the current study are publicly available. The two UC mucosal microarray datasets are available from the Gene Expression Omnibus under accessions GSE87466 (https://www.ncbi.nlm.nih.gov/geo/query/acc.cgi?acc=GSE87466, accessed on 30 August 2026) and GSE75214 (https://www.ncbi.nlm.nih.gov/geo/query/acc.cgi?acc=GSE75214, accessed on 30 August 2026), and the Visium spatial transcriptomic data under accession GSE189184 (sections B5, B10, B12, and C5). The independent validation cohorts used during revision are GSE92415 (UC mucosa, *n* = 183) and GSE39582 (colorectal cancer, *n* = 573 with survival annotation). TCGA-COAD RNA-seq and clinical data (483 tumour and 41 normal samples) were obtained through TCGAbiolinks from the Genomic Data Commons (https://portal.gdc.cancer.gov (accessed on 30 August 2026)) using the STAR-Counts workflow. cis-eQTL summary statistics are available from the eQTLGen consortium (whole blood, *n* = 31,684; https://www.eqtlgen.org (accessed on 30 August 2026)), and colorectal cancer GWAS summary statistics from the IEU OpenGWAS database under accession ebi-a-GCST90018808 (6581 cases and 463,421 controls; https://gwas.mrcieu.ac.uk (accessed on 30 August 2026)). The accessions listed here correspond exactly to those cited in the Methods section. All analysis code, including the scripts for the analyses added during revision, is available at https://github.com/sunwenhao2025-beep/Rscripts-for-Article (accessed on 30 August 2026) and archived at Zenodo under DOI 10.5281/zenodo.22166808 (accessed on 30 August 2026). Correspondence and requests for materials should be addressed to the corresponding author.

## Supplementary Materials

The following supporting information can be downloaded at: https://www.mdpi.com/article/10.3390/biomedicines14092120/s1, Figure S1: Scale-free topology fit and mean connectivity across candidate soft-threshold powers for the UC and TCGA-COAD networks. The power selected for each network is the smallest value at which the scale-free topology model fit exceeds 0.65; Figure S2: LASSO logistic regression in the harmonized UC cohort. (a) Binomial deviance under 10-fold cross-validation as a function of log(lambda); points are the mean and vertical bars the standard error. (b) Number of non-zero coefficients across the same lambda path. Dashed line, lambda.min; dotted line, lambda.1se, the penalty used for feature selection, at which 10 variables are retained; Figure S3: Funnel plots for the two-sample Mendelian randomization analyses of *CXCL1* and *S100P*. Points are individual instruments; the vertical line is the inverse-variance weighted estimate. No asymmetry is apparent for either exposure; Figure S4: Correlations between hub gene expression and the individual pathway signature scores underlying the six therapy signatures summarized in Figure 6c; Figure S5: (a) Kaplan–Meier curves for each hub gene individually in TCGA-COAD, with patients split at the median expression of that gene, (b) omogram for 1-, 3-, and 5-year overall survival fitted on the complete-case subset (*n* = 279, 54 deaths), combining the CXCL1-based risk score with age, sex, and AJCC stage, (c) Calibration curves for the nomogram at 1, 3, and 5 years. The diagonal is perfect calibration; Figure S6: Spatial transcriptomic quality control for the four Visium sections: number of counts, number of detected features, and mitochondrial read fraction per spot, before and after filtering; Figure S7: Spot-level expression distributions of the four hub genes in each of the four sections, shown separately so that within-section spread can be compared with the between-section differences; Table S1: Bootstrap selection frequency and compartment assignment for the candidate genes. Selection frequency is the proportion of 1000 bootstrap resamples of the harmonized UC cohort in which a gene was selected simultaneously by LASSO, random forest, and SVM-RFE. Nearest hub is the reported panel gene with which the candidate correlates most strongly in the harmonized UC matrix, and r is that correlation. Genes of the reported panel are shown in bold; Table S2: Mendelian randomization instrument strength, statistical power, and cis-eQTL availability. *k* is the number of instruments surviving clumping; *R*^2^ is the proportion of expression variance they explain, computed as *Z*^2^/(*N* + *Z*^2^); F statistics are given per SNP and overall. OR at 80% power is the smallest odds ratio per standard deviation of genetically predicted expression detectable at 80% power against 6581 cases and 463,421 controls. The cis-eQTL columns give the number of variants available at three thresholds; *THY1* and *TRIM29* have none at any threshold and could not be tested; Table S3: Section-level linear mixed model of hub gene expression in the Visium data, with the naive spot-level comparison shown for contrast. The model is expression ~ group + (1|section), fitted with lme4 and Satterthwaite degrees of freedom. The naive *p* value treats each spot as an independent observation and is reported only to show the magnitude of the pseudoreplication artefact; it is not the basis of any claim; Table S4: Co-localization coefficients computed separately within each ulcerative colitis section. Values are Spearman correlations between hub gene expression and the cell-type module score across the spots of that section. Consistency of sign between the two sections, rather than a *p* value computed across non-independent spots, is the criterion used in the main text; Table S5: Single-gene results in the two independent cohorts. GSE92415 gives the direction of differential expression in ulcerative colitis mucosa, its Wilcoxon *p* value, and the area under the curve for that gene alone. GSE39582 gives the hazard ratio per standard deviation for overall survival, its 95% confidence interval, *p* value, and the corresponding C-index.
