# Supplementary material for: Multi-Omics Integration Identifies Epithelial-Stromal-Immune Co-Regulators Bridging Ulcerative Colitis and Colorectal Cancer
Source: Biomedicines. 2026 Sep 19;14(9):2120. doi: 10.3390/biomedicines14092120 (PMC13604762; doi:10.3390/biomedicines14092120)
Supplement: Supplementary file 1 [file biomedicines-14-02120-s001.zip › biomedicines-4502317-Supplementary-Materials.pdf]

Supplementary Materials

Multi-Omics Integration Identifies Epithelial-Stromal-Immune Co-Regulators Bridging Ulcerative Colitis and Colorectal Cancer

Wen-hao Sun and Zhi-wei Jiang

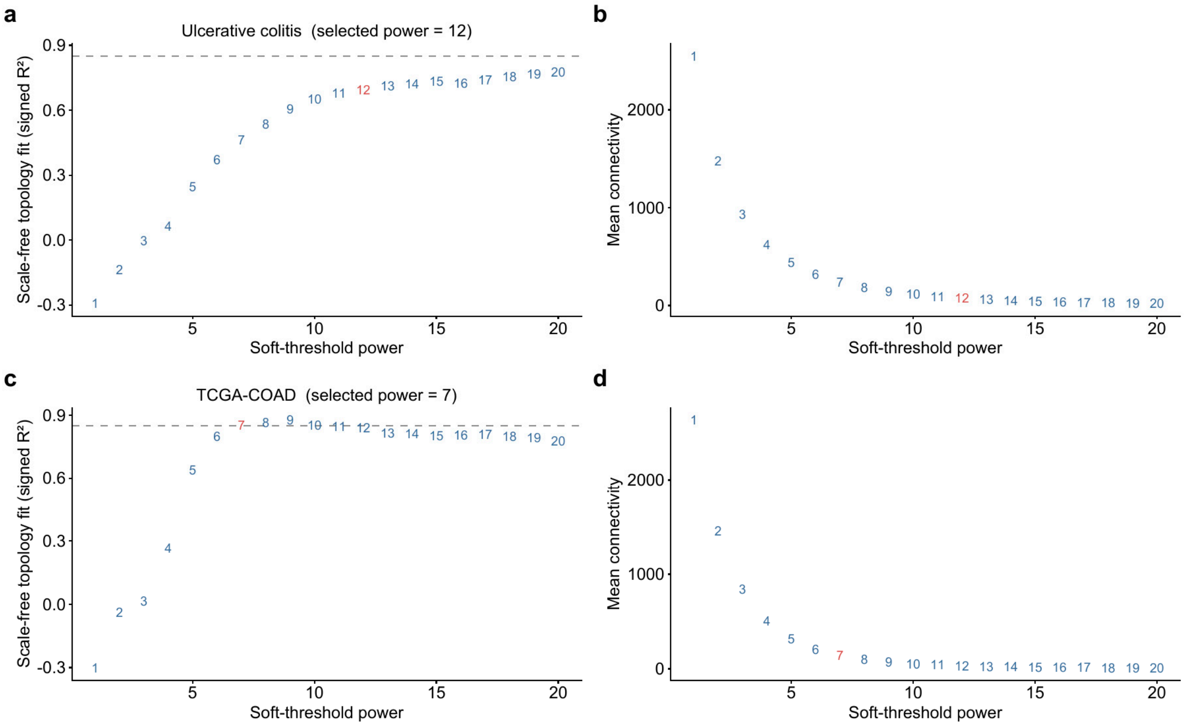

Figure S1. Scale-free topology fit and mean connectivity across candidate soft-threshold powers for the UC and TCGA-COAD networks. The power selected for each network is the smallest value at which the scale-free topology model fit exceeds 0.65.

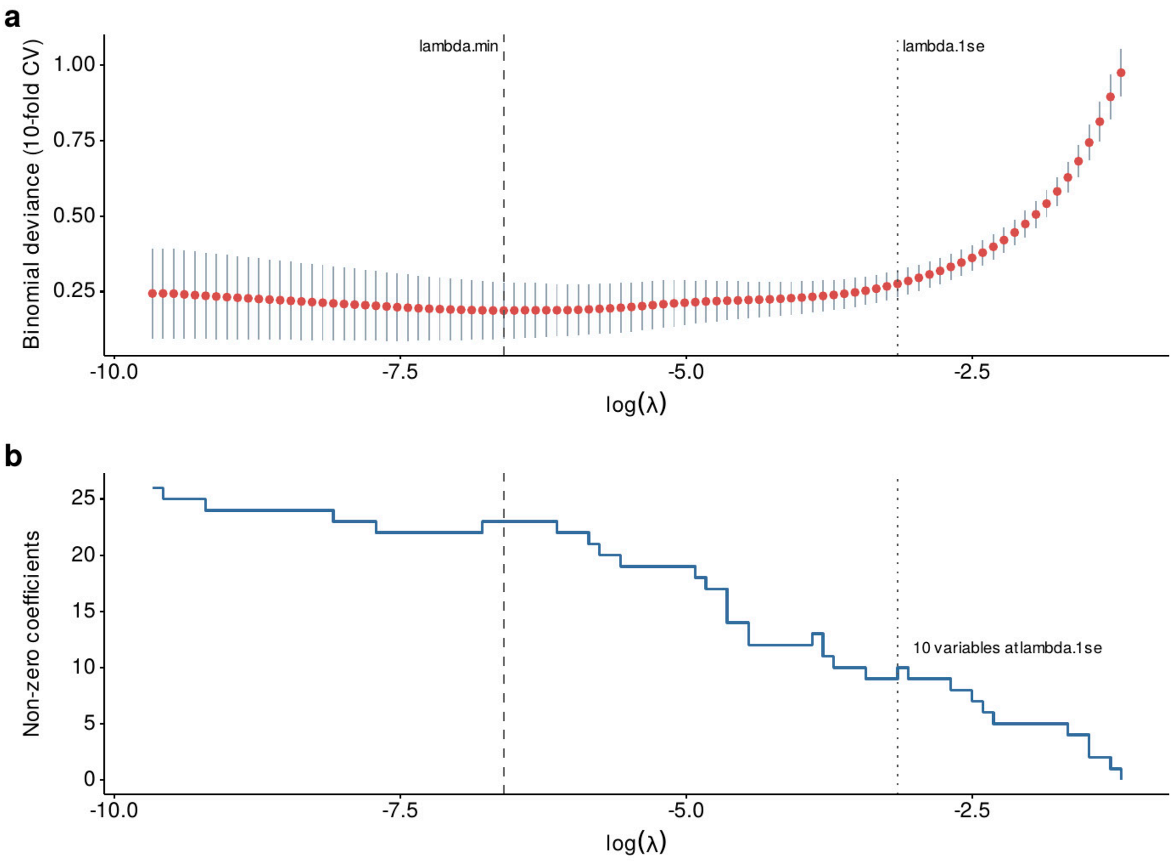

Figure S2. LASSO logistic regression in the harmonized UC cohort. (**a**) Binomial deviance under 10-fold cross-validation as a function of log(lambda); points are the mean and vertical bars the standard error. (**b**) Number of non-zero coefficients across the same lambda path. Dashed line, lambda.min; dotted line, lambda.1se, the penalty used for feature selection, at which 10 variables are retained.

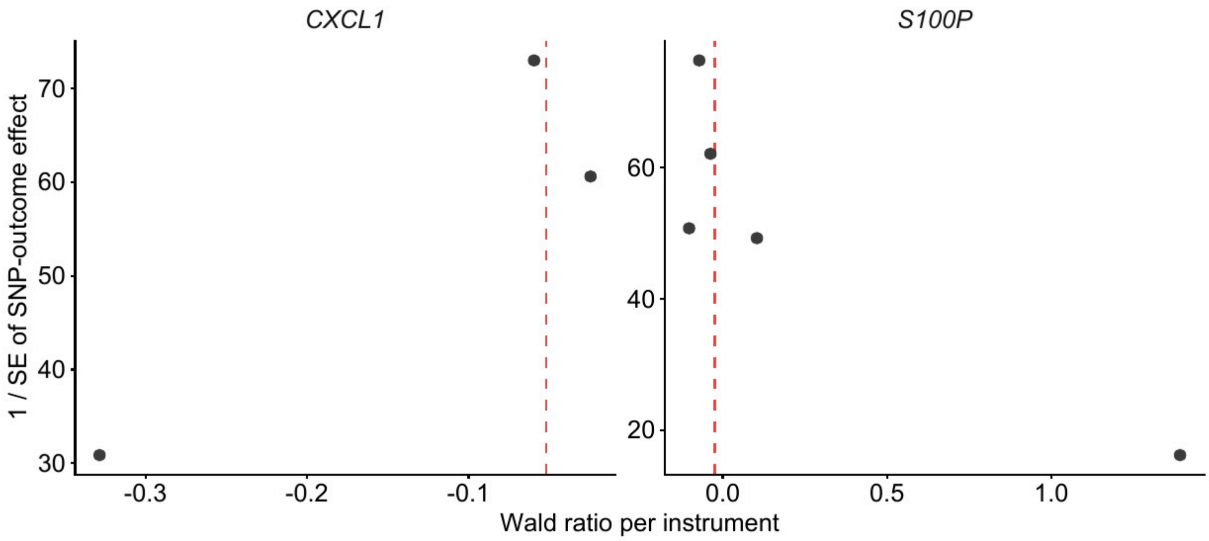

Figure S3. Funnel plots for the two-sample Mendelian randomization analyses of *CXCL1* and *S100P*. Points are individual instruments; the vertical line is the inverse-variance weighted estimate. No asymmetry is apparent for either exposure.

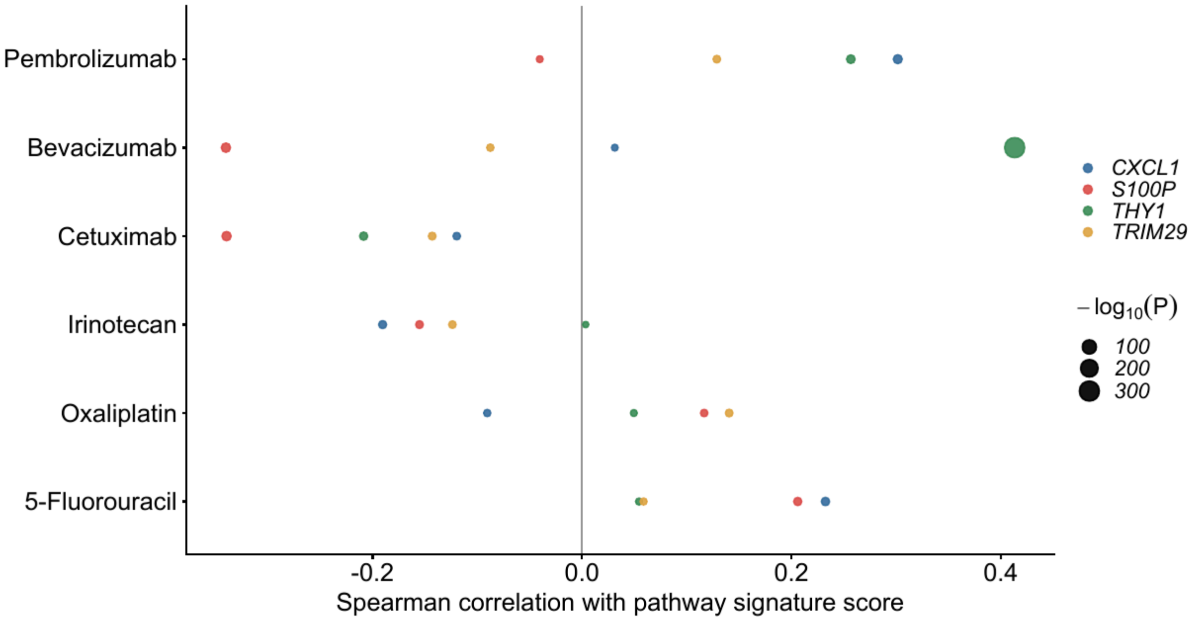

Figure S4. Correlations between hub gene expression and the individual pathway signature scores underlying the six therapy signatures summarized in Figure 6c.

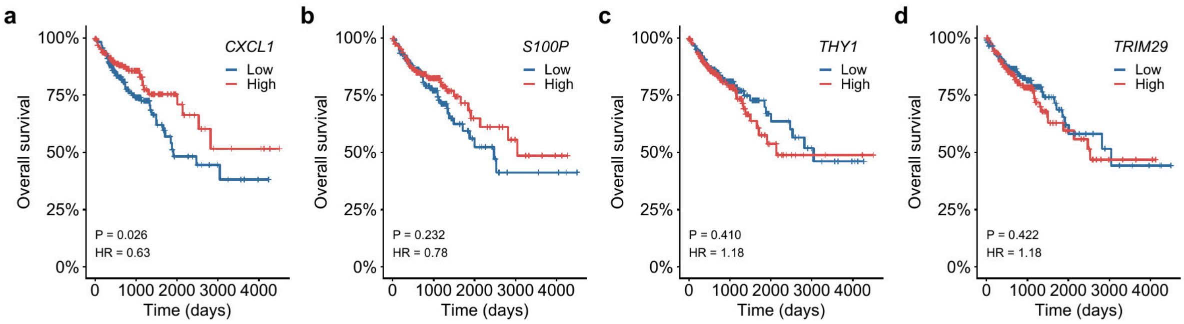

Figure S5a. Kaplan–Meier curves for each hub gene individually in TCGA-COAD, with patients split at the median expression of that gene.

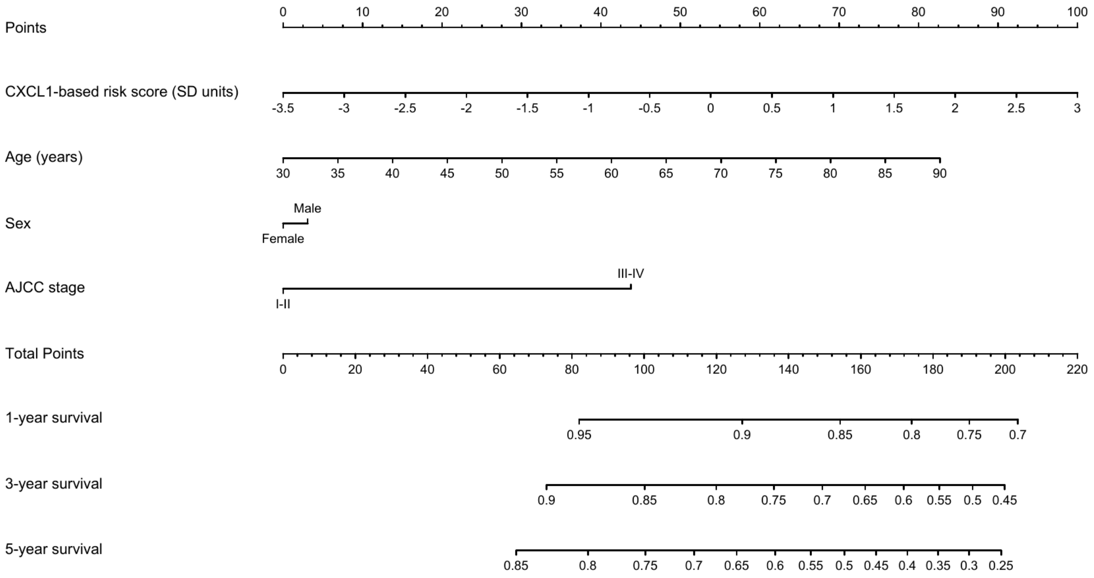

Figure S5b. Nomogram for 1-, 3-, and 5-year overall survival fitted on the complete-case subset (n = 279, 54 deaths), combining the *CXCL1*-based risk score with age, sex, and AJCC stage.

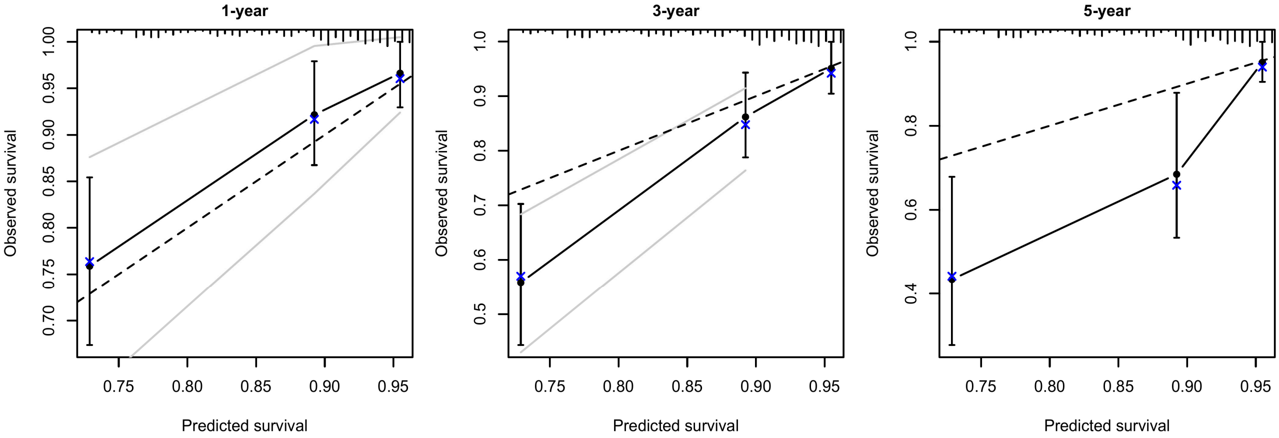

Figure S5c. Calibration curves for the nomogram at 1, 3, and 5 years. The diagonal is perfect calibration.

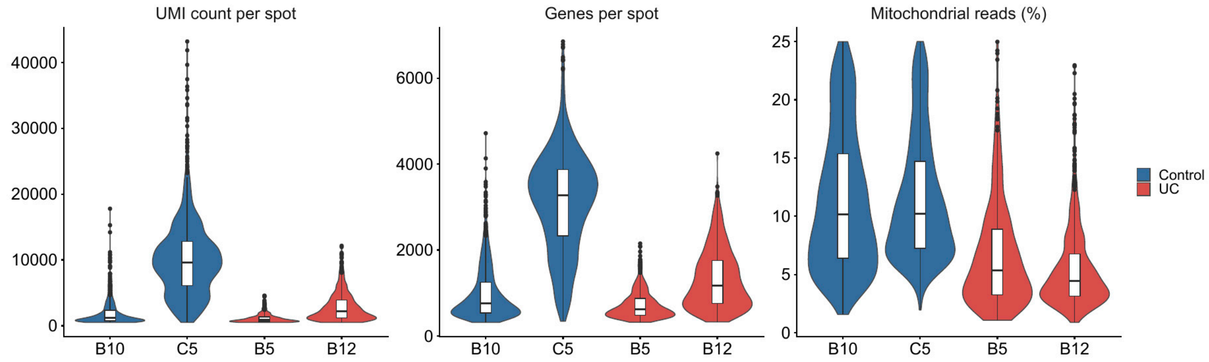

Figure S6. Spatial transcriptomic quality control for the four Visium sections: number of counts, number of detected features, and mitochondrial read fraction per spot, before and after filtering.

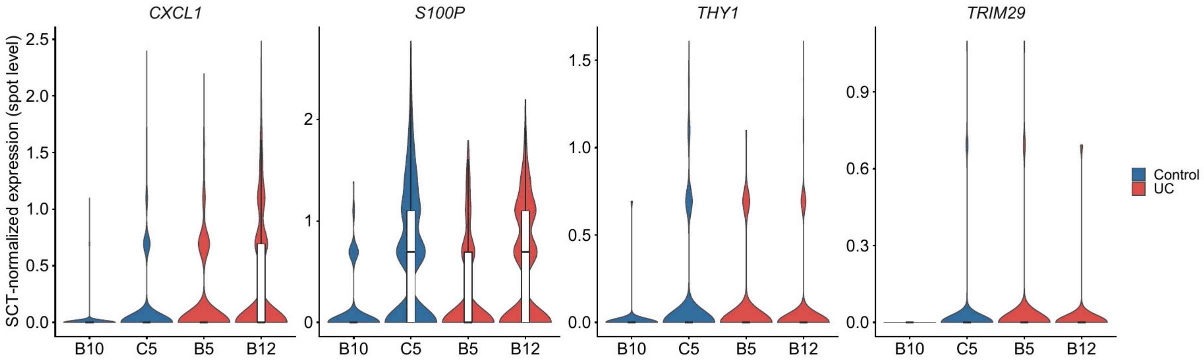

Figure S7. Spot-level expression distributions of the four hub genes in each of the four sections, shown separately so that within-section spread can be compared with the between-section differences.

Table S1. Bootstrap selection frequency and compartment assignment for the candidate genes. Selection frequency is the proportion of 1000 bootstrap resamples of the harmonized UC cohort in which a gene was selected simultaneously by LASSO, random forest, and SVM-RFE. Nearest hub is the reported panel gene with which the candidate correlates most strongly in the harmonized UC matrix, and r is that correlation. Genes of the reported panel are shown in bold.

Gene	Selection frequency	Nearest hub gene	r to nearest hub
S100P	90.1%	*S100P*	1.00
TRIM29	52.2%	*TRIM29*	1.00
DPP10	47.3%	*S100P*	−0.49
LCN2	47.3%	*TRIM29*	0.67
THY1	45.2%	*THY1*	1.00
SCUBE2	24.8%	*THY1*	−0.44
B4GALNT2	22.7%	*S100P*	−0.40
CDH3	17.5%	*TRIM29*	0.82
PRRX1	16.6%	*THY1*	0.88
COL1A2	13.3%	*THY1*	0.88
PCDH17	10.3%	*THY1*	0.86
NPY1R	9.5%	*S100P*	−0.26
CPXM1	6.0%	*THY1*	0.85
IFITM2	5.9%	*CXCL1*	0.84
CXCL1	5.4%	*CXCL1*	1.00
MT1H	4.3%	*S100P*	−0.47
COL3A1	3.5%	*THY1*	0.81
VLDLR	3.3%	*S100P*	−0.37
MYEOV	3.1%	*S100P*	0.74
DEFB1	2.5%	*S100P*	−0.17
CXCL3	2.0%	*CXCL1*	0.90
IL1B	1.6%	*CXCL1*	0.87
HAPLN3	0.9%	*THY1*	0.86
SPARC	0.8%	*THY1*	0.82
MMP9	0.7%	*THY1*	0.87
PNLIPRP2	0.6%	*S100P*	−0.40
BMP3	0.4%	*S100P*	−0.48
CDH11	0.3%	*THY1*	0.79
MT1G	0.2%	*S100P*	−0.49
PDE6A	0.2%	*S100P*	−0.40
PLAU	0.2%	*CXCL1*	0.85
CCL20	0.1%	*CXCL1*	0.55
CXCL2	0.1%	*CXCL1*	0.90
FADS1	0.1%	*THY1*	0.86
LRP8	0.1%	*CXCL1*	0.79
MMP12	0.1%	*CXCL1*	0.79
STC1	0.1%	*CXCL1*	0.82

Table S2. Mendelian randomization instrument strength, statistical power, and cis-eQTL availability. *k* is the number of instruments surviving clumping; R^2 is the proportion of expression variance they explain, computed as $Z^2/(N + Z^2)$; *F* statistics are given per SNP and overall. OR at 80% power is the smallest odds ratio per standard deviation of genetically predicted expression detectable at 80% power against 6581 cases and 463,421 controls. The cis-eQTL columns give the number of variants available at three thresholds; *THY1* and *TRIM29* have none at any threshold and could not be tested.

Gene	k	R^2 (%)	*F* per SNP	*F* overall	OR at 80% power	cis-eQTL, FDR < 0.05	cis-eQTL, $p < 5 \times 10^{-8}$	cis-eQTL, $p < 5 \times 10^{-6}$
CXCL1	3	3.27%	64–546	345	1.21	888	723	856
S100P	5	28.42%	36–8674	2423	1.07	610	510	566
THY1	0	—	—	—	Not testable	0	0	0
TRIM29	0	—	—	—	Not testable	0	0	0

Table S3. Section-level linear mixed model of hub gene expression in the Visium data, with the naive spot-level comparison shown for contrast. The model is expression ~ group + (1 | section), fitted with lme4 and Satterthwaite degrees of freedom. The naive p value treats each spot as an independent observation and is reported only to show the magnitude of the pseudoreplication artefact; it is not the basis of any claim.

Gene	Estimate	SE	df	*t*	*p* (mixed model)	*p* (naive, spot-level)
CXCL1	0.185	0.089	2	2.07	0.174	5.67×10^{-67}
S100P	0.029	0.272	2	0.11	0.926	1.53×10^{-5}
THY1	0.01	0.055	2.02	0.18	0.876	0.252
TRIM29	0.013	0.013	2.09	0.96	0.433	0.003

Table S4. Co-localization coefficients computed separately within each ulcerative colitis section. Values are Spearman correlations between hub gene expression and the cell-type module score across the spots of that section. Consistency of sign between the two sections, rather than a p value computed across non-independent spots, is the criterion used in the main text.

**Gene**	**Compartment**	***r* (section B5)**	***r* (section B12)**	**Same sign**
CXCL1	Epithelium	0.41	0.23	Yes
CXCL1	CAF	−0.11	0.15	No
CXCL1	Neutrophil	0.04	0.12	Yes
CXCL1	Macrophage	−0.08	0.27	No
CXCL1	Treg	−0.05	−0.04	Yes
CXCL1	IFN response	0.32	0.32	Yes
S100P	Epithelium	0.62	0.41	Yes
S100P	CAF	−0.27	0.02	No
S100P	Neutrophil	0.03	−0.02	No
S100P	Macrophage	−0.08	0.13	No
S100P	Treg	−0.1	−0.09	Yes
S100P	IFN response	0.32	0.27	Yes
THY1	Epithelium	−0.26	−0.05	Yes
THY1	CAF	0.2	0.14	Yes
THY1	Neutrophil	0.04	0.03	Yes
THY1	Macrophage	0.15	0.04	Yes
THY1	Treg	−0.04	0.04	No
THY1	IFN response	−0.01	0.02	No
TRIM29	Epithelium	0.2	0.13	Yes
TRIM29	CAF	−0.1	0.02	No
TRIM29	Neutrophil	−0.04	0.06	No
TRIM29	Macrophage	0	0.09	Yes
TRIM29	Treg	0.02	0.03	Yes
TRIM29	IFN response	0.14	0.11	Yes

Table S5. Single-gene results in the two independent cohorts. GSE92415 gives the direction of differential expression in ulcerative colitis mucosa, its Wilcoxon p value, and the area under the curve for that gene alone. GSE39582 gives the hazard ratio per standard deviation for overall survival, its 95% confidence interval, p value, and the corresponding C-index.

Gene	GSE92415 direction	GSE92415 p	GSE92415 AUC	GSE39582 HR per SD	95% CI	GSE39582 p	GSE39582 C-index
CXCL1	Higher in UC	1.00×10^{-11}	0.957	0.892	0.77–1.03	0.114	0.54
S100P	Higher in UC	8.87×10^{-12}	0.958	0.984	0.85–1.14	0.833	0.499
THY1	Higher in UC	1.03×10^{-11}	0.957	1.116	0.96–1.29	0.142	0.512
TRIM29	Higher in UC	1.25×10^{-12}	0.977	1.107	0.96–1.28	0.168	0.535
